# Colossal Cryogenic Electro‐Optic Response Through Metastability in Strained BaTiO_3_ Thin Films

**DOI:** 10.1002/adma.202507564

**Published:** 2025-10-11

**Authors:** Albert Suceava, Sankalpa Hazra, Aiden Ross, Ian Reed Philippi, Dylan Sotir, Brynn Brower, Lei Ding, Yingxin Zhu, Zhiyu Zhang, Himirkanti Sarkar, Saugata Sarker, Yang Yang, Suchismita Sarker, Vladimir A. Stoica, Darrell G. Schlom, Long‐Qing Chen, Venkatraman Gopalan

**Affiliations:** ^1^ Department of Materials Science and Engineering The Pennsylvania State University University Park PA 16802 USA; ^2^ Department of Physics The Pennsylvania State University University Park PA 16802 USA; ^3^ Platform for the Accelerated Realization, Analysis, and Discovery of Interface Materials (PARADIM) Cornell University Ithaca NY 14853 USA; ^4^ Department of Materials Science and Engineering Cornell University Ithaca NY 14853 USA; ^5^ Department of Engineering Science and Mechanics The Pennsylvania State University University Park PA 16802 USA; ^6^ Cornell High Energy Synchrotron Source Cornell University Ithaca NY 14853 USA; ^7^ Department of Nuclear Engineering The Pennsylvania State University University Park PA 16802 USA; ^8^ Materials Research Institute The Pennsylvania State University University Park PA 16802 USA; ^9^ Kavli Institute at Cornell for Nanoscale Science Ithaca NY 14853 USA; ^10^ Leibniz‐Institut für Kristallzüchtung Max‐Born‐Straße 2 12489 Berlin Germany

**Keywords:** barium titanate, electro‐optic effect, phase‐field method, pockels effect, strain‐tuning

## Abstract

The search for thin film electro‐optic materials that can retain superior performance under cryogenic conditions has become critical for quantum computing. Barium titanate thin films show large linear electro‐optic coefficients in the tetragonal phase at room temperature, which is severely degraded down to ≈200 pm V^−1^ in the rhombohedral phase at cryogenic temperatures. There is immense interest in manipulating these phase transformations and retaining superior electro‐optic properties down to liquid helium temperature. Utilizing the thermodynamic theory of optical properties, a large low‐temperature electro‐optic response is designed by engineering the energetic competition between different ferroelectric phases, leading to a low‐symmetry monoclinic phase with a massive electro‐optic response. The existence of this phase is demonstrated in a strain‐tuned BaTiO_3_ thin film that exhibits a linear electro‐optic coefficient of 2516 ± 100 pm V^−1^ at 5 K, which is an order of magnitude higher than the best reported performance thus far. Importantly, the electro‐optic coefficient increases by 100 × during cooling, unlike the conventional films, where it degrades. Further, at the lowest temperature, significant higher order electro‐optic responses also emerge. These results represent a new framework for designing materials with property enhancements by stabilizing highly tunable metastable phases with strain.

## Graphical abstract

Utilizing the thermodynamic theory of optical properties, a colossal cryogenic electro‐optic response in BaTiO_3_ thin films is designed and demonstrated by stabilizing a low symmetry metastable monoclinic phase via epitaxial strain tuning with an electro‐optic response reaching ≈2516 pm V^−1^ at 5 K. This approach represents a new paradigm for engineering large property enhancements in materials applied toward cryogenic photonics.

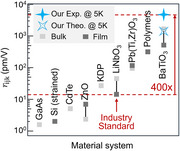

## 1 Introduction

The electro‐optic (EO) effect in lithium niobate crystals enables our internet by encoding electrical to optical signals.^[^ ^1^, ^2^, ^3^ ^]^ The EO effect describes the change in refractive index, *n*, of a material due to an applied electric field, *E_k_*, given by $\Delta \;{\left(\frac{1}{n^{2}}\right)}_{ij}=r_{ijk}\;E_{k}+s_{ijkl}E_{k}E_{l}$ in nonmagnetic materials, where *r_ijk_* is the Pockel tensor representing the linear effect, *s_ijkl_* is the Kerr tensor representing the quadratic effect, and the dummy subscripts indicate crystal physics axes. This allows for the phase, amplitude, or polarization of light to be modulated by a driving electrical signal. Electro‐optic materials have recently re‐emerged as a key technology in the field of quantum computing, where the EO effect can be leveraged to perform microwave‐to‐optical transduction.^[^ ^4^, ^5^ ^]^ Qubits based on Josephson junctions and trapped ions utilize microwave frequencies to write and read quantum states at cryogenic temperatures.^[^ ^6^, ^7^, ^8^, ^9^ ^]^ Electro‐optic materials are required for the transduction of these microwave signals (operating at millikelvin temperature) to communicate with infrared light that is the standard for optical networks (operating at room temperature). Cryogenic electro‐optics is also important for on‐chip quantum photonic circuits, trapped ion quantum computing schemes, and developments in low‐temperature science.^[^ ^5^, ^10^, ^11^, ^12^, ^13^, ^14^ ^]^ Nonetheless, a materials gap exists, namely EO materials operating at cryogenic temperatures with low thermal budget and a small footprint directly integrated on a chip and with electro‐optic coefficients of *r_eff_* > 1000 pm V^−1^. No such materials currently exist. Barium titanate (BaTiO_3_) appears promising because of its large *r*_51_ ≈ 1640 pm V^−1^ at room temperature; however the best literature reported value for films of BaTiO_3_ is *r_eff_* ≈ 200 pm V^−1^ at 4 K.^[^ ^14^, ^15^ ^]^ In these prior studies, the temperature‐dependent performance of the device revealed a nearly 3 × reduction in *r_eff_* following cooling from room to cryogenic temperature, owing to the occurrence of multiple ferroelectric–ferroelectric phase transitions. Attempts to suppress or overcome these transitions have not yielded significant improvements thus far. With regards to its electrical properties, the microwave dielectric properties of BaTiO_3_ ceramics prepared under various methods have been extensively characterized, with the dielectric function generally rolling off above 10 GHz with an accompanying increase in loss.^[^ ^16^, ^17^, ^18^, ^19^, ^20^, ^21^, ^22^, ^23^ ^]^ Regardless, existing examples of electro‐optic modulators based on thin film BaTiO_3_ have promisingly demonstrated a consistent response at cryogenic temperatures under modulation frequencies up to 30 GHz, leaving the low‐temperature functional optical properties as a significant area for improvement.^[^ ^14^ ^]^

Many high‐performance piezoelectric materials are discovered at morphotropic phase boundaries, namely regions where compositional tuning forces a competition between distinct structural phases, yielding an intermediate low symmetry phase with superior properties relative to the parents.^[^ ^24^, ^25^, ^26^, ^27^, ^28^ ^]^ Similarly, across thermotropic phase boundaries in BaTiO_3_ and KNbO_3_ single crystals, low symmetry monoclinic phases are stabilized by local strain and fields generated within frustrated domain microstructures created by cycling across thermal phase transitions.^[^ ^29^ ^]^ In particular, the optical second harmonic generation coefficients in BaTiO_3_ single crystals were enhanced by up to 4.4 × relative to the bulk values, and by 2.3 × in KNbO_3_ in metastable monoclinic phases stabilized by inhomogeneous strain and fields. One strategy to engineer large nonlinear optical property enhancements is thus to stabilize metastable phases, but homogeneously over the entire sample.

We demonstrate this in BaTiO_3_ thin films, a material recognized for having one of the largest electro‐optic coefficients in its tetragonal (T) phase at room temperature in its bulk form and thus being at the forefront of the search for cryogenic electro‐optic materials.^[^ ^15^, ^30^, ^31^, ^32^ ^]^ Using phase‐field simulations, we design the optimal epitaxial strain conditions to promote the competition between ferroelectric phases near liquid helium temperatures, resulting in a cryogenic monoclinic (M) phase. The films demonstrate a 10 × improvement in the electro‐optic effect over the best demonstrated values in the literature at 10 K.^[^ ^14^ ^]^ Our findings demonstrate a new paradigm for engineering electro‐optic materials under cryogenic conditions.

## 2 Temperature‐Strain Phase Diagram of BaTiO_3_

**Figure 1:**
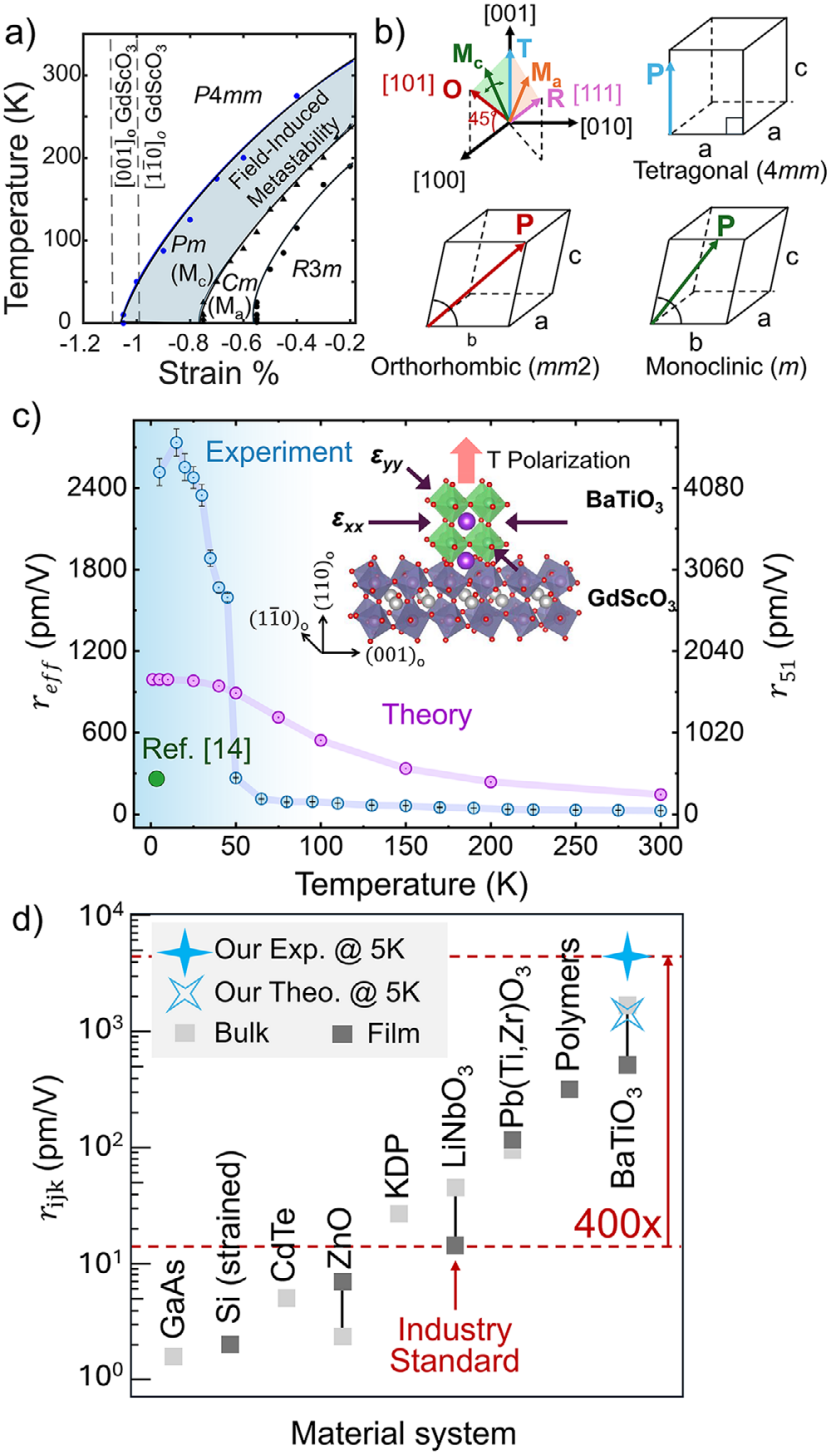
a) Temperature‐strain phase diagram for compressively strained BaTiO_3_ from phase‐field simulations. The blue region indicates a metastable monoclinic phase that may be created by applying an electric field perpendicular to the tetragonal axis and removing it subsequently. b) Representation of polarization directions in tetragonal, orthorhombic, and intermediate monoclinic phases. c) Comparison between experimentally measured (blue) and phase‐field simulation (purple) curves of the effective electro‐optic coefficient responsible for the index change observed under the experimental geometry of Figure 2a. Comparison to the leading cryogenic coefficient found in literature is provided in (green).^[^ ^14^ ^]^ (inset) Schematic of epitaxial BaTiO_3_ under a compressive biaxial strain. d) Comparison between the maximum electro‐optic response obtained in this work and several other benchmark materials.

Bulk BaTiO_3_ undergoes a series of phase transitions from cubic to tetragonal at 130 °C, tetragonal to orthorhombic at 5 °C, and orthorhombic to rhombohedral at −90 °C.^[^ ^33^, ^34^ ^]^ **Figure** **1**a depicts the predicted temperature‐dependent phase diagram of BaTiO_3_ as a function of biaxial epitaxial strain, *ɛ* = (*a*_||_ – *a*_o_)/*a*_o_, where *a*_o_ is the equivalent cubic lattice parameter extrapolated from the high temperature BaTiO_3_ cubic phase (4.008 Å) and *a*_||_ is the in‐plane lattice parameter of the biaxially strained BaTiO_3_. Under a biaxial compressive strain, the tetragonal *c* (space group *P*4*mm*) phase becomes more stable by minimizing its elastic energy as compared to the other ferroelectric phases. As the biaxial compressive strain decreases, an energetic competition between the chemical energy, which determines the intrinsic stability of the ferroelectric phases (favoring the rhombohedral phase at low temperatures), and the elastic energy (favoring the tetragonal *c* phase) emerges. Under certain strain conditions at cryogenic temperatures, this competition results in a compromise between the two energetic components resulting in the monoclinic *a* (M_a_) phase (space group *Cm*), which is a bridging phase between the tetragonal and rhombohedral phase.^[^ ^35^ ^]^ In addition to the thermodynamically stable phases, after applying an electric field and retracting we find that there exists a field‐induced metastable monoclinic phase that extends beyond the equilibrium monoclinic phase boundary, forming a monoclinic *c* phase (M_c_) (space group *Pm*), which acts as a bridging phase between the tetragonal and orthorhombic phase, as depicted in Figure 1b.

As the temperature decreases further, the system approaches the tetragonal (T) to monoclinic (M) phase boundary, where these competing phases are nearly degenerate in energy. In this regime, a small electric field can shift the thermodynamic stability between states inducing a polarization rotation and enabling a large electro‐optic response, as depicted in Figure 1c. By integrating the thermodynamic theory of optical properties in ferroelectrics with phase‐field simulations, we compute the temperature‐dependent electro‐optic response at 1550 nm.^[^ ^36^ ^]^ Our results predict a large enhancement of the linear electro‐optic coefficient, driven by the highly susceptible ferroelectric polarization of the monoclinic phase near the phase boundary. The general behavior may be understood using the thermodynamic theory of the electro‐optic effect:

(1) $$r_{131}=\left(\frac{\partial B_{13}}{\partial E_{1}}\right)≅\left(\frac{\partial B_{13}}{\partial P_{1}^{L}}\right)\;\left(\frac{\partial P_{1}^{L}}{\partial E_{1}}\right)=f_{131}^{L}\;\chi_{11}^{L}$$

where *r_ijk_* is the electro‐optic coefficient, $B_{ij}={\left(\frac{1}{n^{2}}\right)}_{ij}$ is the optical dielectric stiffness defined in terms of the refractive index *n*, $P_{i}^{L}$ is the lattice polarization (the ionic and electronic components of ferroelectric polarization, which arises due to the displacement of the lattice), and *E_i_* is the electric field. We decompose the electro‐optic coefficient into a linear polar‐optic effect $\left(f_{131}^{L}\right)$ and the dielectric susceptibility of the lattice polarization $\left(\chi_{11}^{L}\right)$. The linear polar‐optic effect is proportional to the spontaneous polarization and remains roughly constant down to low temperature in the compressively strained films. Therefore, the enhancement of the electro‐optic effect is primarily driven by the increase of $\chi_{11}^{L}$ near the tetragonal‐to‐monoclinic phase boundary, where the lattice polarization is highly susceptible to rotation within the monoclinic mirror plane *m*.

## 3 The Electro‐Optic Effect in Strained BaTiO_3_

### 3.1 Cryogenic Electro‐Optic Response

To validate this design approach, we characterize the magnitude of the Pockels effect in BaTiO_3_ grown on (110)_o_ GdScO_3_ (subscript “o” for orthorhombic), where lattice mismatch results in a compressive strain of −1.0%. Epitaxial growth of a ≈37 nm film was achieved by using molecular‐beam epitaxy with in‐situ reflection high‐energy electron diffraction (RHEED) to monitor surface quality. As shown in Figures S1 and S2 (Supporting Information), asymmetric reciprocal space maps taken at the GdScO_3_ 332_o_ peak reveal alignment with the BaTiO_3_ 103_t_ (subcript “t” for tetragonal) peak in *Q_x_*, confirming epitaxial growth. *θ‐2θ* X‐ray diffraction scans confirm *c*‐axis out‐of‐plane orientation of the films in the room temperature T phase, with rocking curve measurements demonstrating film peaks with full‐width half‐max measures of 119 arcsec as shown in Figure S3 (Supporting Information). Post growth atomic force microscope scans reveal a root‐mean‐squared surface roughness of 392 pm as shown in Figure S4 (Supporting Information), confirming a smooth film morphology. Piezoelectric force microscopy scans shown in Figure S5 (Supporting Information) failed to provide evidence for antipolar domain structures. High‐resolution scanning transmission electron microscopy images confirm coherent epitaxial growth and high interfacial quality, as shown in Figure S6 (Supporting Information). Additional experimental details on film growth and structural characterization are provided in Section 5.

Due to the compressive strain enforced by the substrate, at room temperature, the BaTiO_3_ films are entirely tetragonal with the polar [001] axis in the out‐of‐plane direction. 500 nm square electrodes of 80 nm Pt/5 nm Ti were lithographically deposited onto the film surface with a separation gap of 200 µm to apply a field in the in‐plane direction, corresponding to the [100] crystallographic direction, with a schematic of this shown in Figures S7 and S8 (Supporting Information). In the T phase of BaTiO_3_, the crystal physics 1, 2, and 3 directions are conveniently aligned with the crystallographic [100], [010], and [001] directions.

**Figure 2:**
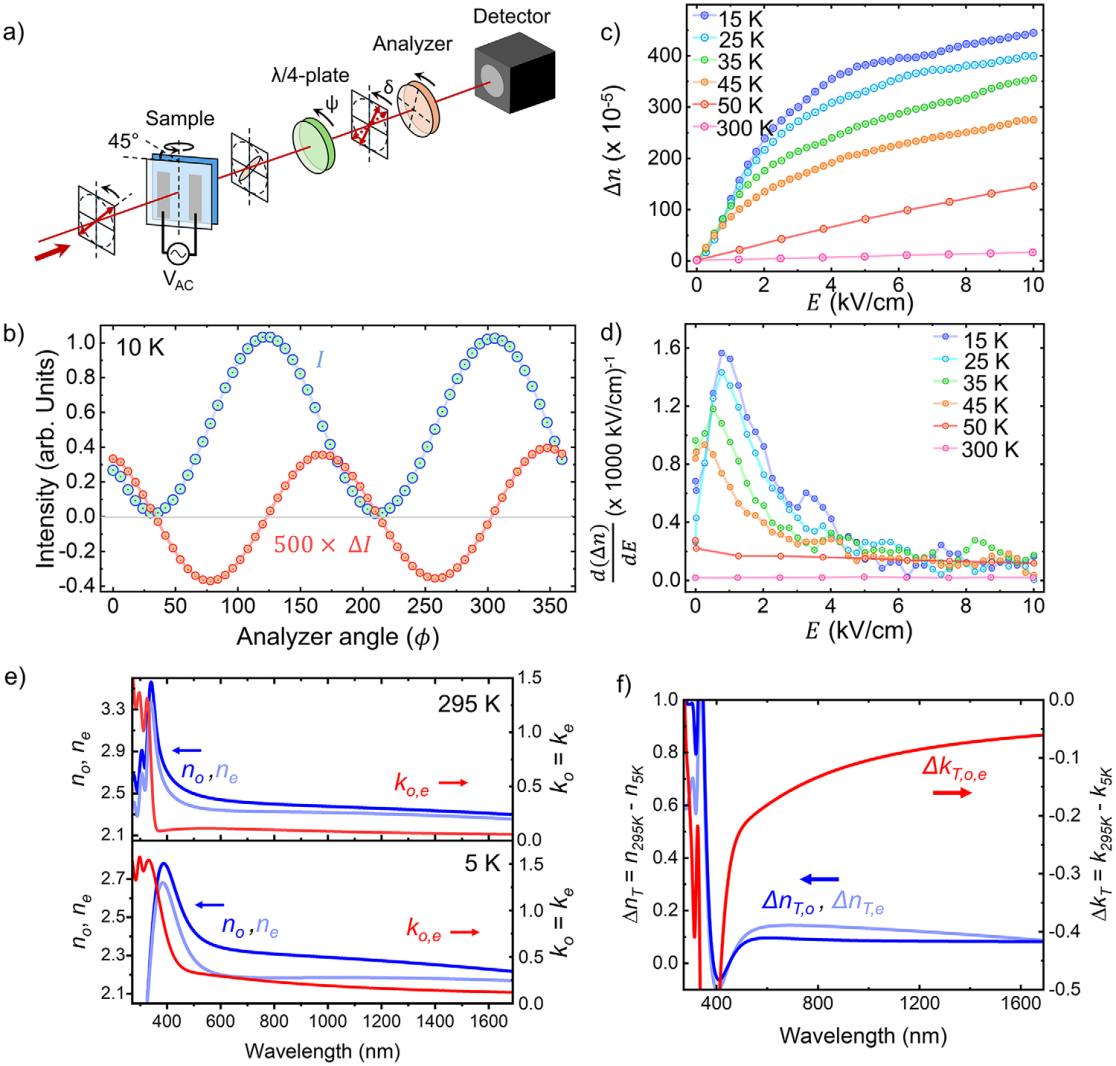
a) Schematic of experimental PCSA setup for measuring the electro‐optic coefficient. b) Experimental unmodulated and modulated intensity curves, displaying the expected π/2 relative phase shift. c) Δ*n* versus applied electric field taken at the analyzer angle which maximizes the modulated intensity at different temperatures. d) The derivative of Δ*n* versus applied electric field curves, showing the offset bias required for maximum modulation efficiency. e) Refractive index and extinction coefficient of strained BaTiO_3_ film versus wavelength, at room temperature and at 5 K, as determined by variable angle ellipsometry. f) Relative refractive index and extinction coefficient changes between room temperature and 5 K, Δ *n_T_* = *n*_295K_  − *n*_5K_, Δ*k*  = *k*_295K_  − *k*_5K_.

Electro‐optic measurements were performed using a polarizer‐sample‐compensator‐analyzer (PSCA) based measurement setup, as shown in **Figure** **2**a.^[^ ^37^, ^38^, ^39^, ^40^, ^41^, ^42^, ^43^, ^44^, ^45^, ^46^, ^47^, ^48^, ^49^, ^50^, ^51^, ^52^ ^]^ In this method, the change in transmission of light through a pair of crossed polarizers as a function of applied electric field is detected to quantify the change in the refractive indices of the sample. Further details on the operating principle are provided in Section 4 and within ref. [37, 38, 39, 40, 41, 42, 43, 44, 45, 46, 47, 48, 49, 50, 51, 52]. A knife‐edge characterization of the probe beam is provided in Figure S9 (Supporting Information). Other measurement methods, such as the Teng–Man approach, were considered, but the PSCA method ultimately pursued due to the restrictions the epitaxial strain condition imposes on the electrode geometry. Confidence in the measurement procedure was established by determining the electro‐optic coefficient of a well‐known LiNbO_3_ reference sample, whose data are included in Figure S10 (Supporting Information).

For characterization of the BaTiO_3_ on GdScO_3_ sample of interest, the incident polarization is set to lie between two principal optic axes of the sample, which in this study are the [100] and [010] in‐plane crystallographic axes of the BaTiO_3_ film. The BaTiO_3_ thin film refractive indices were characterized using spectroscopic ellipsometry at room temperature and at 5 K. The extracted dispersion curves are shown in Figure 2e, and a detailed description of the optical modeling procedure and additional reflection and transmittance measurements performed is provided in Note S3, Figures S11 and S12 (Supporting Information). The ellipsometry measurements also served to refine the film thickness to a value of 36.53 nm, which was used in the subsequent analysis. With an in‐plane electric field *E*_1_, the index ellipsoid of the tetragonal phase distorts following Equation (2), taking the original 1, 2, and 3 principal axes to a new set of axes: 1′, 2′ = 2, 3′:

(2) $$\frac{x^{2}}{n_{1}^{2}}+\frac{y^{2}}{n_{2}^{2}}+\frac{z^{2}}{n_{3}^{2}}+2xz\;r_{51}\;E_{1}=1$$

In the absence of the field *E*_1_, *x* ≡ 1, *y* ≡ 2, *z* ≡ 3 are the principal (Eigen) crystal physics axes, and *n*_1_, *n*_2_, *n*_3_ are the principal refractive indices along those directions with *n*_1_ = *n*_2_  = *n_o_* and *n*_3_ = *n_e_* for the T phase of BaTiO_3_, a negative uniaxial material.^[^ ^1^ ^]^ The action of *r*_51_ (the last term in Equation (2)) would lead to new Eigen coordinates in the *x*‐*z* plane, leading to *x*′ ≡ 1′ and *z*′ ≡ 3′ axes. The *y*′ ≡  2′ =  *y* ≡ 2 would remain unchanged under this field. This point is illustrated in detail in Note S4 and Figure S15 (Supporting Information).

To detect the electro‐optic effect under this geometry, where the *z* ≡ 3 axis is pointed normal to the film surface, the sample was tilted 45° away from the incident probe as depicted in Figure 2a. This results in the probe beam now experiencing two orthogonal refractive indices *n_o_* and *n_e_* (θ) without any external voltage applied to the sample, and *n_o_* ^′^ = *n_o_* and *n_e_* (θ)^′^ with the voltage applied. The birefringence induced by the sample results in a change in the probe polarization from a linear to an elliptical state, resulting in a non‐zero transmission through an analyzer oriented orthogonally to the initial polarization state when a voltage is applied.

The unmodulated transmission function, *I*, of this system without an applied electric field is described $I=I_{0}\;\mathrm{co}s^{2}\left(\phi +\delta \right)$, where, ϕ represents the angle through which the analyzer is rotated and δ represents an overall phase angle accumulated during beam transmission through the sample and other optical components before the analyzer, as shown in Figure 2a. When an applied electric field is applied across the sample, the resulting modulation to the transmission function, Δ*I*, can be expressed with respect to the first order in Δδ as follows:

(3) $$\Delta I=I_{0}\;\Delta \delta \;\sin2\left(\phi +\delta \right)$$

Figure 2b shows the experimentally measured *I* and Δ*I* curves at 10 K for the BaTiO_3_ on GdScO_3_ sample fitted to the above equations. The derived Δδ can be converted into the electric‐field induced birefringence, $\Delta n=\frac{\Delta \delta \lambda }{2\pi L}$, where, λ  =  1550 nm is the wavelength of light used for measurements, and *L* is the distance light travelled through the BaTiO_3_ film given by *L*  =  *t*/cos θ_*film*_, where *t* is the film thickness and θ_*film*_ is the angle describing the direction of propagation of light inside the film layer, considering the 45° probe angle of incidence and Snell's law and assuming a single pass. The effective electro‐optic coefficient, 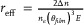 is then calculated, where *E*  is the amplitude of the AC bias applied to the sample to measure the modulated transmission function, Δ*I* and *n_e_* (θ_*film*_) is the refractive index seen by p‐polarized light, described at length in Note S4 (Supporting Information). Following Equation (3), the experimentally measured maximum value for the modulated transmission function, Δ*I*, is obtained at the analyzer angle that results in the halfway point of the unmodulated transmission function, *I*, which is where the slope is maximized resulting in a $\frac{\pi }{2}$ phase shift between the two curves. A linear dependence in the maximum modulated intensity plotted against the applied electric field above 50 K (Figure 2c), confirms the origin of the response as the Pockels effect. The nonlinear behavior that emerges below 50 K is discussed in the following sections. This measurement is performed at several discrete temperatures to characterize the low‐temperature properties of the sample, with the compensator position, ψ, optimized to cancel out the native birefringence of the sample at every temperature. The effective electro‐optic coefficient observed, *r_eff_*, is converted into the tensor coefficient *r*_51_ through a geometrical analysis of the modulated index ellipsoid, as described in Note S4 (Supporting Information).

The unmodulated refractive index was directly measured at room temperature and at 5 K. For the analysis described in the preceding section, the refractive index measured at 5 K was used to analyze electro‐optic data collected below 50 K, while the room temperature index was used to interpret data collected above 50 K. The separation between data sets at 50 K is chosen to reflect the phase transition that occurs at that temperature, as seen in the electro‐optic measurements and second harmonic generation data to follow. An analysis of the electro‐optic response using a linear interpolation of these refractive index values as opposed to a step function is presented in Figure S14 (Supporting Information). With the effective electro‐optic coefficient expressed in full as $r_{eff\;}=\frac{2}{n_{e}{\left(\theta_{film}\right)}^{3}E}\;\left(\frac{\Delta I\;\cdot \;\lambda }{I_{0}\;\cdot \;2\pi L}\right)$, the collective error can be expressed as the sum of the errors of all terms in the products weighed by their exponents: $\frac{dr_{eff\;}}{r_{eff\;}}=3\left(\frac{dn_{o}}{n_{o}}\right)+\frac{dE}{E}+\frac{d\Delta I}{\Delta I}+\frac{dI_{0}}{I_{0}}+\frac{dL}{L}$. In other words, every percent error in the unmodulated refractive index will propagate a three times larger percent error in the extracted electro‐optic coefficient. Assuming that the refractive index in the temperature range between 5 and 295 K lies between the two endpoints measured, this results in a maximum error in the index equal to the difference between the two values, which is an error of 3.4%. Thus, the maximum error in the determination of the electro‐optic coefficients due to inaccuracy in the index interpolation becomes 10.2%. An increase in the extinction coefficient of the film at low temperature may arise from the transition from a unipolar tetragonal structure to a multidomain monoclinic structure with potentially four domain variants, which might promote additional scattering of light from the domain walls.^[^ ^53^, ^54^ ^]^

As shown in Figure 1c, the measured effective electro‐optic coefficient reaches a peak value of 2735 ± 100 pm V^−1^ at a temperature of 15 K, representing over a 100 × enhancement from the room temperature value of 25 ± 2 pm V^−1^. This is in direct contrast with previous results in the literature, where the electro‐optic coefficients of relaxed BaTiO_3_ films are reduced to nearly a third of their room temperature value at similar temperatures.^[^ ^14^ ^]^ It is important to acknowledge that the room temperature electro‐optic coefficient of the strained BaTiO_3_ is lower than that of relaxed films grown on Si found in literature.^[^ ^31^ ^]^ Phase‐field simulations provide support for this trade‐off being an intrinsic effect of the strained condition. There also exists the possibility of the grown film possessing oppositely oriented tetragonal *c* domains resulting in a reduction in the observed electro‐optic effect. Piezoelectric force microscopy was performed to image such potential antipolar domains, but no such domains were revealed, Figure S5 (Supporting Information). Nevertheless, the strained condition leads to superior performance at low temperatures. The reproducibility of this response is discussed in Note S5, Figure S17, and Table S3 (Supporting Information) with regards to both thermal and electrical cycling. While the sample appears to be remarkably robust and consistent with regards to electrical cycling, some variation in the nonlinear response is observed in every instance the sample is cooled. Most noteworthy is a reduction in the largest observed *r*_*eff*_ after the sample had undergone several cooling‐heating cycles, suggesting that thermal hysteresis and the initial configuration of the domain microstructure at low temperature carries significant implications for the nonlinear electro‐optic response. The large enhancement at cryogenic temperatures arises from the emergence of the M_c_ phase as discussed next.

### 3.2 Nonlinearity in the Cryogenic Electro‐Optic Response

**Figure 3:**
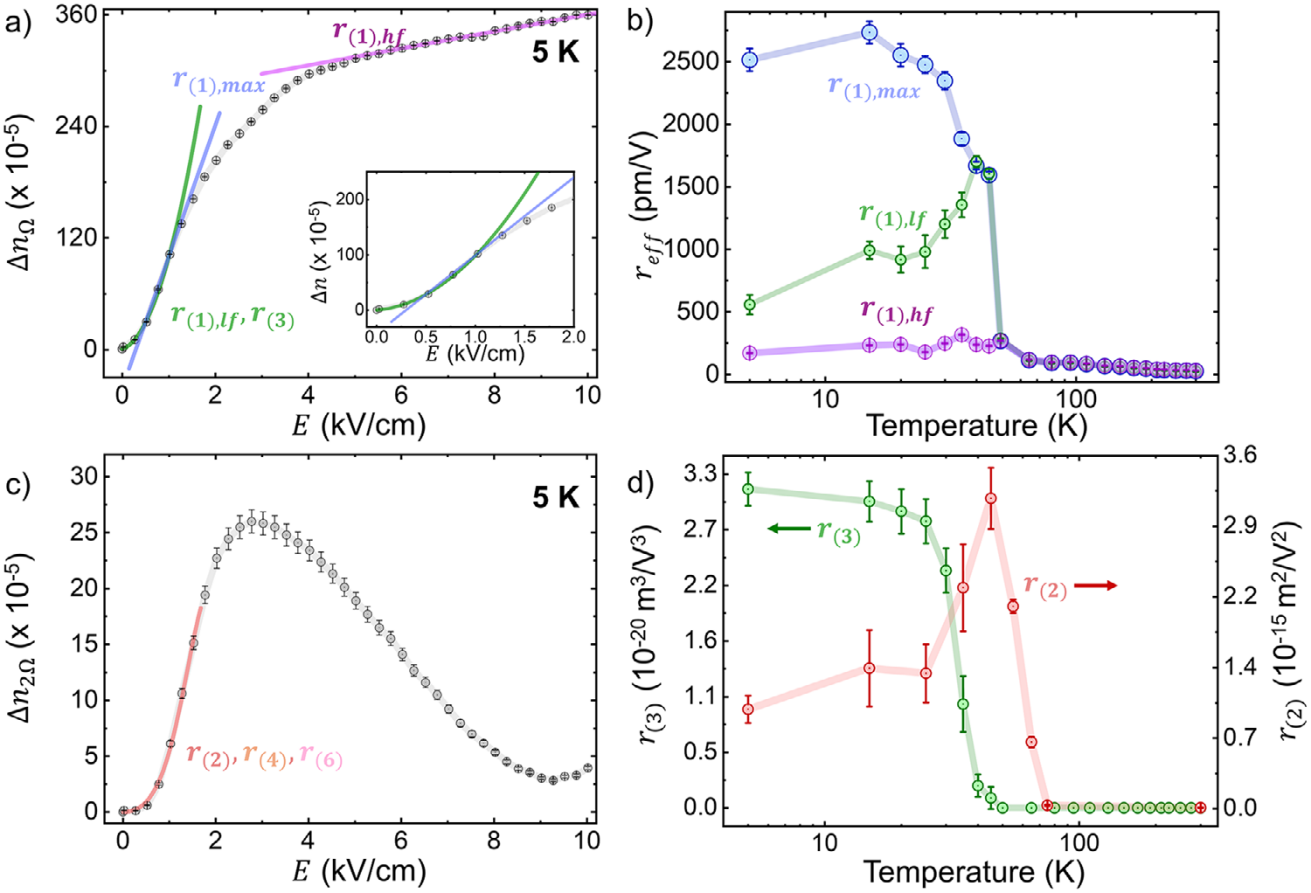
a) Various interpretations of the effective electro‐optic response *r_eff_*, depending on which part of the nonlinear response is of interest. b) *r*_(1),*max*_, *r*_(1),*hf*_, and *r*_(1),*lf*_ as a function of temperature. c) Observed electro‐optic response recorded at double the modulation frequency, 2Ω, as a function of temperature, indicating a nonlinear response d) Higher order electro‐optic coefficients *r*_(2)_ and *r*_(3)_ extracted from polynomial fits of the nonlinear 2 Ω and Ω frequency responses, respectively.

The field dependence of the electro‐optic response exhibits a striking difference below 50 K from that above (Figure 2c), where a field‐dependent nonlinearity is observed. Below 50 K, a knee in the curves becomes apparent, separating a linear regime at high fields from a much stronger linear response at low fields. Below 25 K, a second knee emerges at very low fields roughly below 0.5 kV cm^−1^, resulting in an S‐curve‐like shape as shown in **Figure** **3**a. Response curves of the predicted monoclinic phase can thus be discussed in terms of three different regimes: the low‐field limit, where the index change appears strictly nonlinear, a mid‐range high‐slope linear regime containing the point of inflection for the curve, and a lower‐slope high‐field linear regime.

An examination of the derivative of Δ*n* as a function of *E* curves (Figure 2d) reveals that the slope of the high‐field regime converges to a common value, which corresponds to an *r_eff_* = *r*_(1),*hf*_  ≈ 200 pm V^−1^, the temperature dependence of which is displayed in Figure 3b. The derivative of Δ*n* as a function of *E* also reveals the exact point of inflection of the response curves located in the intermediate voltage range, where the magnitude of the linear electro‐optic response is maximized. By taking the linear slope at the inflection point, one can define a second value for *r_eff_* = *r*_(1),*max*_ , depicted in Figure 3a. It is this value of *r_eff_* that is presented in Figure 1c. From an engineering perspective, *r*_(1),*max*_ can be accessed by applying a small DC bias offset alongside AC modulation to maximize the sensitivity of the electro‐optic response. This DC bias would be equivalent to the point where the derivative of Δ*n* as a function of *E* is maximized, as shown in Figure 2d. For response curves below 35 K, the low field regime can be fit by a higher‐order polynomial function in odd powers.

The choice to only attempt a fit with odd‐order polynomial terms can be understood by considering the higher‐order expansion of the electro‐optic effect under an AC field: $\Delta \;{\left(\frac{1}{n(t)}\right)}^{2}=\sum_{n}r_{\left(n\right)}E{\left(t\right)}^{n}$ where (*n*) denotes the *n^th^* order electro‐optic effect (*r*_(*n*)_ is not to be confused with *r_ij_* the linear electro‐optic tensor coefficient). For a sinusoidal field the electro‐optic response becomes: $-\Delta n\left(t\right)\;\frac{2}{n^{3}}=\sum_{n}r_{\left(n\right)}{E_{0}}^{n}\cos^{n}\left(\Omega t\right)$, where cos ^*n*^(Ω*t*) can be expanded as:

(4) $$\begin{array}{c} \cos^{n}\left(\Omega t\right)=\frac{1}{2^{n-1}}\;\sum_{k=\frac{n}{2}+1}^{n}\left(\begin{array}{c} n\\ k \end{array}\right)\cos\left[\left(2k-n\right)\Omega t\right]+\frac{1}{2^{n}}\left(\begin{array}{c} n\\ n/2 \end{array}\right)\;\mathrm{for}\;\mathrm{even}\;n \end{array}$$

(5) $$\begin{array}{c} \cos^{n}\left(\Omega t\right)=\frac{1}{2^{n-1}}\;\sum_{k=\frac{n}{2}+1}^{n}\left(\begin{array}{c} n\\ k \end{array}\right)\cos\left[\left(2k-n\right)\Omega t\right]\;\mathrm{for}\;\mathrm{odd}\;n \end{array}$$

It follows then that:

(6) $$\begin{array}{c} -\Delta n\left(t\right)\;\frac{2}{n^{3}}=\left(\frac{1}{2}r_{\left(2\right)}{E_{0}}^{2}+\frac{3}{8}r_{\left(4\right)}{E_{0}}^{4}+\frac{5}{16}r_{\left(6\right)}{E_{0}}^{6}+\cdot \cdot \cdot \right)\;\\ +\left(r_{\left(1\right)}E_{0}+\frac{3}{4}r_{\left(3\right)}{E_{0}}^{3}+\frac{5}{8}r_{\left(5\right)}{E_{0}}^{5}+\cdot \cdot \cdot \right)\cos\left(\Omega t\right)\\ +\left(\frac{1}{2}r_{\left(2\right)}{E_{0}}^{2}+\frac{1}{2}r_{\left(4\right)}{E_{0}}^{4}+\frac{15}{32}r_{\left(6\right)}{E_{0}}^{6}+\cdot \cdot \cdot \right)\cos\left(2\Omega t\right)\\ +\left(\frac{1}{4}r_{\left(3\right)}{E_{0}}^{3}+\frac{5}{16}r_{\left(5\right)}{E_{0}}^{5}+\cdot \cdot \cdot \right)\cos\left(3\Omega t\right)\\ +\left(\frac{1}{8}r_{\left(4\right)}{E_{0}}^{4}+\frac{3}{16}r_{\left(6\right)}{E_{0}}^{6}+\cdot \cdot \cdot \right)\cos\left(4\Omega t\right) \end{array}$$

It can be seen then that when detecting an electro‐optic response at the same frequency as the driving field, only odd‐powered higher order terms should manifest in the observed signal as experimentally detected by a lock‐in amplifier.

A fit of up to third‐order terms is sufficient for the low field regime of the electro‐optic response recorded at the first harmonic of the driving electric field, with the resultant third‐order electro‐optic coefficients *r*_(3)_ shown as a function of temperature in Figure 3d. Equation (6) also reveals that an intrinsically nonlinear material response is expected to yield a signal at detection frequencies that are higher harmonics of the driving electric field. Specifically, the presence of a quadratic electro‐optic effect should be detected when locking on to twice the modulation frequency and integer multiples thereof. In order to confirm the nonlinear nature of the material response at cryogenic temperatures, Δ*n* versus *E* curves were also measured at several discrete temperatures while locking on to double the frequency of the modulation field, 2Ω, with a representative result shown in Figure 3c and curves obtained at other temperatures shown in Figure S18b (Supporting Information). A fit of up to 6th order terms yields very good agreement with the data at low fields. We note that to fit the response curve for the full range of voltages applied, yet even higher order terms are required. The extracted quadratic electro‐optic coefficients *r*_(2)_ are provided in Figure 3d, while the temperature dependence of the higher order terms *r*_(4)_ and *r*_(6)_ is provided in Figure S18a (Supporting Information). A phenomenological model based on the Avrami equation is also capable of providing an approximate fit to the nonlinear response, as shown in Figure S19 (Supporting Information). This model is further discussed in Note S6 (Supporting Information).

Comparing different methods of defining the linear *r_eff_* as *r*_(1),*max*_, *r*_(1),*hf*_, and *r*_(1),*lf*_ reveals a convergence above 50 K when the nonlinearity in the response curves vanishes. The *r*_(1),*lf*_ converges to *r*_(1),*max*_ with increasing temperature, while at low temperatures it approaches *r*_(1),*hf*_, suggesting that as the S‐curve character grows more prominent, the low‐field regime may mirror the high‐field regime. The relationship between the Pockels *r_eff_* and individual tensor elements are discussed in detail in Note S4 (Supporting Information), which includes a discussion on all possible tensor element contributions to the observed response and experiments conducted in an attempt to isolate the dominant coefficients. Following these considerations and supported by phase field simulations shown in Figure S16 (Supporting Information), the *r_eff_* is found to primarily reflect the intrinsic *r*_51_ Pockels coefficient reduced by a factor of ≈1.7 ×, yielding a maximum value of *r*_51_ =  4649 ± 170 pm V^−1^ at 15 K, as shown in Figure 1c.

The quadratic response observed at 2 Ω is found to maximize ≈50 K where the nonlinear response initially sets in. The fitted response is found to be within the same order of magnitude as *s*_11_ −  *s*_12_ = *r*_(2),11_  − *r*_(2),12_ measures of the observed quadratic electro‐optic effect in bulk BaTiO_3_ (*s_ij_* being the more common notation for the quadratic electro‐optic effect as opposed to *r*_(2),*ij*_), and several orders of magnitude higher than the response of other classical nonlinear optical materials like KH_2_PO_4_ (KDP).^[^ ^1^, ^55^ ^]^ We note that while the observed Δ*n*_2Ω_ continues to increase with decreasing temperature, this does not manifest as an increase in the extracted *r*_(2)_, but rather in the higher order *r*_(4)_ and *r*_(6)_ terms required to yield a good fit of the complex response (Figure S18, Supporting Information). Further exploration of the full extent of this nonlinearity with regards to even higher harmonic responses and extraction of higher order electro‐optic coefficients will be investigated in subsequent works.

The nonlinearity, which is primarily responsible for the cryogenic property enhancement as defined through *r*_(1),*max*_ appears to be correlated to the emergence of the metastable monoclinic structure. For applied fields below the saturation regime, a continuous monoclinic distortion can be produced through a “swaying” of the ferroelectric polarization vector away from the tetragonal out‐of‐plane *c*‐axis, with an in‐plane component developing in the direction of the applied field. This results in a large lattice dielectric susceptibility and large electro‐optic response. Nonetheless, polarization rotation eventually saturates near 21° (as predicted by phase‐field simulation) and the lattice susceptibility is reduced leading to the high‐field regime.^[^ ^56^ ^]^

### 3.3 Understanding the M_c_ Phase Through Optical Second‐Harmonic Generation

**Figure 4:**
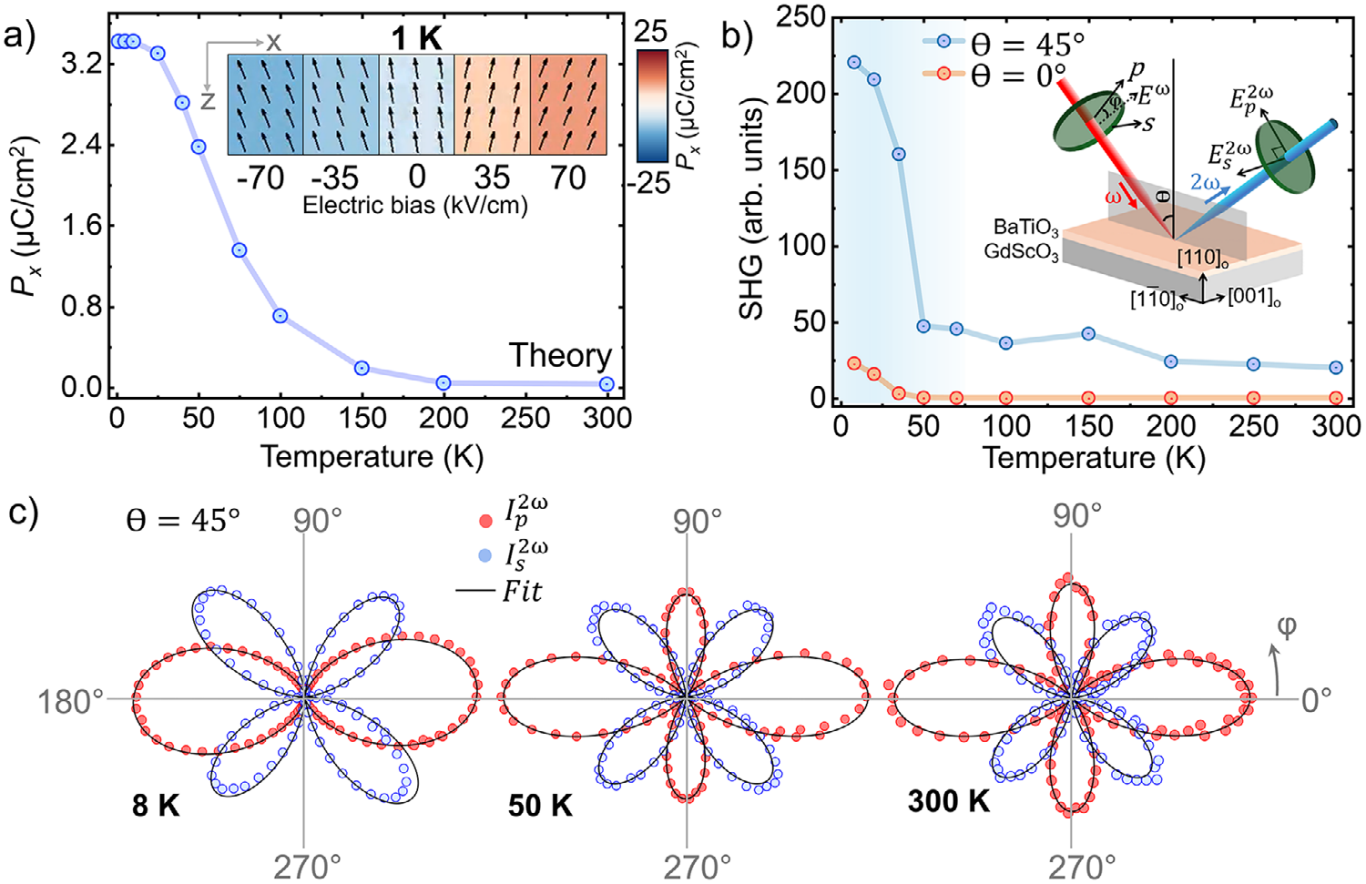
a) Phase‐field simulated values of the in‐plane polarization, *P_x_*, as a function of temperature, reflecting the degree of monoclinic distortion. (inset) Phase field simulated polarization vectors under applied field at 1 K, showcasing the susceptibility of the lattice. b) SHG signal versus temperature at θ = 0° and θ = 45° incidence angle measurement conditions, for an input fundamental polarization, φ, along 0°. c) SHG polarimetry taken at θ = 45° incidence angle and at 300, 50, and 8 K, indicating tetragonal 4*mm* symmetry at and above 50 K and a monoclinic *m* symmetry below 50 K. The solid lines are theory fits as described in Note S7 (Supporting Information).

To confirm the presence of a new monoclinic phase below 50 K, complementary second‐harmonic generation (SHG) polarimetry measurements were performed (see Section 5: Experimental Section). The SHG process describes the generation of light at frequency 2*ω* when light of frequency ω passes through a non‐centrosymmetric medium following the third‐rank tensor equation: $P_{i}^{2\omega }=\varepsilon_{o}\;d_{ijk}E_{j}^{\omega }E_{k}^{\omega }$, where $E_{j}^{\omega }$ and $E_{k}^{\omega }$ are optical electric fields at frequency ω and polarization directions *j* and *k*, $P_{i}^{2\omega }$ is the radiating nonlinear polarization generated in the material at frequency 2ω and polarization direction *i*, with efficiency described by the SHG tensor coefficient *d_ijk_* of the material, and ε_*o*_ the permittivity of vacuum.^[^ ^2^ ^]^ Owing to its nature as a third‐rank tensor property similar to the Pockels effect, polar phases and transitions can be observed with extreme sensitivity.^[^ ^57^ ^]^ At normal incidence, no SHG was observed at room temperature as expected for the tetragonal 4*mm* phase. The observation of a non‐zero SHG signal in this geometry would explicitly confirm a reduction in symmetry of the film structure. Indeed, the normal incidence SHG (**Figure** **4**b) is zero until 50 K, confirming a transition to a lower symmetry structure. This is consistent with the prediction of the in‐plane ferroelectric polarization component, *P_x_*, as a function of temperature (Figure 4a). SHG polarimetry performed at θ = 45° incidence (Figure 4c) and its modeling (see Note S7 and Table S4, Supporting Information) indicate a tetragonal 4*mm* phase until 50 K followed by a monoclinic *m* structure with four domain variants arising from the positive and negative shear in each of the two mirror planes perpendicular to *x* and to *y*.^[^ ^58^ ^]^ To further support the interpretation of the *r*_(1)*max*_ response as due to a monoclinic distortion under an external electric field, the electric‐field‐dependent SHG response is also measured and shown in Figure S20b–e (Supporting Information). Under an external field consistent with that applied during electro‐optic experiments, the polarimetry curves continue to evolve in a way that requires a monoclinic *m* model to fit, suggesting that additional monoclinic distortions occur in the softened, low‐temperature lattice. Hysteresis in the samples was observed by performing both electro‐optic measurements under a DC bias and SHG measurements under DC bias. Both yield minimal discernible hysteretic behavior, with the results shown in Figure S21 (Supporting Information). These findings suggest that the metastable monoclinic phase enables a continuous, repeatable transformation of the polarization vector.

Additional low‐temperature X‐ray characterization could serve as explicit confirmation of the suspected cryogenic monoclinic phase and will be investigated in future work. Both SHG and X‐ray characterization confirmed an analogous metastable phase in strained KNbO_3_ thin films, thus strengthening the SHG‐based claims presented for BaTiO_3_.^[^ ^58^ ^]^

## 4 Conclusion

Through theory and experiments, we demonstrate that strain can be used to engineer intermediate low‐symmetry phases in BaTiO_3_ thin films, where a cryogenic metastable monoclinic phase exhibits a large linear electro‐optic response, as well as a large nonlinear EO response. Due to the competition between multiple ferroelectric phases with similar thermodynamic stability, an emergent low‐symmetry phase can be stabilized. This “bridging phase” manifests as a monoclinic structure, which is noteworthy for the ability of its lattice to deform in the presence of an applied electric field. The freedom for the polar axes to sway away from the tetragonal [001] *c*‐axis with an in‐plane component in the (001) plane results in a large dielectric susceptibility and a large effective electro‐optic effect, the highest reported thus far. Future work is required to measure this EO response at GHz frequencies where it is of technological importance. Nonetheless, this work illustrates the power of symmetry breaking and stabilizing new metastable states in thin films toward achieving superior properties that are otherwise not available in the equilibrium phases.

## 5 Experimental Section

### Growth of BaTiO_3_ on GdScO_3_ Thin Films

The sample was grown in a Veeco GEN10 MBE system equipped with an epiray GmbH THERMALAS laser substrate heater, a 1 kW CO_2_ laser with a wavelength of 10.6 µm that irradiates the backside of the substrate over a circular area with a diameter of ≈14 mm. Barium (Sigma–Aldrich, 99.99% purity) was supplied using a conventional differentially pumped effusion cell, and titanium using a Veeco Ti‐Ball source. One monolayer of BaO was deposited first, after which the sources were simply co‐deposited. The barium flux was ≈5 × 10^13^ atoms (cm^2^ × s)^−1^ and the titanium flux was ≈1 × 10^13^ atoms (cm^2^ × s)^−1^, i.e., a Ba:Ti flux ratio of 5:1. The barium flux was determined by a quartz crystal microbalance, and the titanium flux by X‐ray reflectivity of a calibration film of BaTiO_3_ grown on an SrTiO_3_ (001) substrate at *T_sub_* = 1200 °C, with an oxidant pressure of 1 × 10^−6^ Torr of O_2_ + 10% O_3_ and a Ba:Ti ratio of 5:1. The BaTiO_3_ film was grown on a GdScO_3_ (110)_o_ substrate (CrysTec GmbH), to a thickness of ≈36 nm.

Ozone was used as the oxidant at a background pressure of 1×10^−6^ Torr of O_2_ + 10% O_3_. The film was cooled in the same oxidant and pressure in which it was grown to *T*_sub_ < 200 °C before the oxidant was turned off. The substrate temperature, measured by a pyrometer operating at 7.5 µm on the backside of the substrate during growth, was 1160 °C. Due to the high substrate temperature, the vapor pressure of barium‐containing species over BaTiO_3_, chiefly BaO, was significantly higher than that of titanium‐containing species over BaTiO_3_, so excess barium will desorb from the surface leaving behind a single‐phase BaTiO_3_ film within an adsorption‐controlled growth window. As GdScO_3_ absorbs well at 10.6 µm, the backside of the substrate was not coated.

X‐ray diffraction (XRD) and reciprocal space mapping (RSM) analysis were done with a Panalytical Empyrean X‐ray diffractometer using Cu *K*_α1_ radiation. Atomic force microscopy (AFM) was performed using an Asylum Cypher ES Environmental AFM. RHEED images were taken during growth using a Staib electron source operating at 14 kV.

### High Resolution Scanning Transmission Electron Microscopy

A sample of the BaTiO_3_ on GdScO_3_ film was prepared for high‐resolution scanning transmission electron microscopy (HR‐STEM) using focused ion beam (FIB) lift‐out, followed by sequential milling at 30, 16, 5 kV, and a final cleaning at 2 kV to progressively reduce surface damage. Scanning transmission electron microscopy (STEM) and energy‐dispersive X‐ray spectroscopy (EDX) analysis were performed using a ThermoFisher Scientific Titan TEM operated at 300 kV, spot size of 6, camera length of 115 mm, C2 aperture of 70 µm, convergence angle of 25.2 mrad. HR‐STEM was collected with a beam current of 0.07 nA and improved by performing drift correction on sequentially acquired STEM images, followed by image averaging. The resulting images were denoised using a LASSO‐like soft‐thresholding filter to suppress high‐frequency noise while preserving structural features. The EDX results show the mass percent distribution maps of individual elements.

### Polarization‐Based Electro‐Optic Measurement

Electro‐optic measurements were performed using a homebuilt PSCA setup with a 1550 nm HP 81689A continuous‐wave laser source and a Nirvana 2017 balanced detector. For absolute intensity measurements, a mechanical chopper was placed in the laser path and modulated at 1.54 kHz for detection with a Stanford SR830 lock‐in amplifier. For measuring the modulated transmission intensity, an Agilent 33220A signal generator amplified by a Trek 610C high voltage amplifier was used to apply a 555 Hz sine wave to the sample, with the lock‐in amplifier reference signal changed to the function generator reference. The nonlinear electro‐optic response was verified by also collecting electro‐optic modulation signal at double the frequency of the driving voltage, i.e., at 1110 Hz. Samples were cooled to liquid He temperatures using a Janis 30 continuous flow cryostat system.

The polarization of the probe was set to lie between the ordinary and extraordinary refractive indices, as depicted in Figure 2a. An analyzer placed at the end of the setup was oriented orthogonal to the initial probe polarization state, resulting in no transmission of light through the setup when the sample and compensator were not included. With the sample introduced, the transmission becomes nonzero on account of the birefringence‐induced ellipticity. A quarter‐wave compensator was then added immediately after the sample to cancel out the native material birefringence with no voltage applied. This returns the transmission of the setup to zero until a voltage is applied to modulate the sample refractive indices and reintroduce ellipticity to the probe.

### Second‐Harmonic Generation Polarimetry

Second‐harmonic generation polarimetry experiments were performed using a fundamental wavelength of 800 nm generated by a Spectra‐Physics Solstice Ace amplified Ti:Sapphire laser with a 1 kHz repetition rate and 100 fs pulse width. Light was focused onto the sample using a 10 cm focal length lens, producing a 50 µm diameter spot size as determined by knife‐edge measurements. Samples were cooled to liquid He temperatures using a Janis 30 continuous flow cryostat system. A bare GdScO_3_ substrate of the same type produces no appreciable SHG signal under the same incident power, and thus the SHG signal measured was interpreted as originating entirely from the strained BaTiO_3_ film.

### Phase‐Field Simulations

Based on the thermodynamic theory of optical properties, a phase‐field model might formulated, where the thermodynamics of the ferroelectric system was described by its free energy functional,

(7) $$\begin{array}{c} F=\int \left[f^{L}\left(T,\;P_{i}^{L}\right)+f^{e}\left(T,\;P_{i}^{e}\right)+f^{L-e}\left(P_{i}^{L},\;P_{i}^{e}\right)+f^{grad}\left(\nabla_{i}P_{j}^{L}\right)\right.\\ +\left.f^{elas}\left(P_{i}^{L},\;P_{i}^{e},\;\varepsilon_{ij}\right)+f^{elec}\left(P_{i}^{L},\;P_{i}^{e},\;E_{i}\right)\right]dx^{3} \end{array}$$

where $P_{i}^{L}\left(x_{i},\;t\right)$ is the lattice polarization, $P_{i}^{e}\left(x_{i},\;t\right)$ is the electrical polarization, and σ_*ij*_(*x*,*t*) is the stress.^[^ ^36^ ^]^

The evolution of the lattice polarization and electronic polarization is solved separately. The lattice polarization is computed following the relaxational approximation, where relaxational kinetics for the lattice polarization was assumed, and the mechanical displacement, electrical potential, and electrical polarization instantaneously reach equilibrium, leading to the time‐dependent Ginzburg–Landau equation

(8) $$\gamma_{ij}^{L}\;\frac{\partial P_{j}^{L}}{\partial t}=-\frac{\delta F}{\delta P_{i}^{L}}$$

where $-\frac{\delta F}{\delta P_{i}^{L}}$ is the thermodynamic driving force for the temporal evolution of the lattice polarization. The local stress and lattice polarization distribution was used as inputs to the electronic polarization dynamic equation

(9) $$\mu_{ij}^{e}\frac{\partial^{2}P_{j}^{e}}{\partial t^{2}}+\gamma_{ij}^{e}\;\frac{\partial P_{j}^{e}}{\partial t}=-\frac{\delta F}{\delta P_{i}^{e}}$$

where $-\frac{\delta F}{\delta P_{i}^{e}}$ is the thermodynamic driving force for the temporal evolution of the electronic polarization. To compute the local electronic dielectric susceptibility, Equation (7) was solved assuming a small periodic electric field is applied, which yields an analytical solution

(10) $${\tilde{\chi }}_{\mathrm{ij}}^{e,1}\left(x_{i},\;\omega \right)={\left[B_{\mathrm{ij}}^{e}\left(x_{i}\right)-\varepsilon_{0}\left(i\omega \gamma_{\mathrm{ij}}^{e}+\omega^{2}\mu_{\mathrm{ij}}^{e}\right)\right]}^{-1}\;$$

which is related to the curvature of the free‐energy landscape with respect to the electronic polarization by, $B_{\mathrm{ij}}^{e}\;\left(x_{i}\right)=\varepsilon_{0}\;\left(\frac{\partial^{2}f}{\partial P_{i}^{e}\partial P_{j}^{e}}\right)$. Solving Equation (14) (assuming λ =  1550 nm) the local refractive indices might be computed, from the local lattice polarization and stress distribution, thereby naturally including the electro‐optic effect and potential piezo‐optic effects. As the lattice polarization distribution evolves in response to an applied electric field, the corresponding change to the electronic dielectric susceptibility and the refractive index was computed.

Here $f^{L}\left(P_{i}^{L}\right)$ describes the intrinsic stability of the lattice polarization compared to the high symmetry phase (*m* $\bar{3}$ *m*) as a Taylor expansion of the polarization about the high symmetry phase, this was equivalent to the Landau free energy density:

(11) $$\begin{array}{ccc} f^{L}\;\left(T,\;P_{i}^{L}\right) & = & f_{o}+a_{ij}\left(T\right)P_{i}^{L}P_{j}^{L}+a_{ijkl}P_{i}^{L}P_{j}^{L}P_{k}^{L}P_{l}^{L}+a_{ijklmn}P_{i}^{L}P_{j}^{L}P_{k}^{L}P_{l}^{L}P_{m}^{L}P_{n}^{L}+\\ & & a_{ijklmnop}P_{i}^{L}P_{j}^{L}P_{k}^{L}P_{l}^{L}P_{m}^{L}P_{n}^{L}P_{o}^{L}P_{p}^{L} \end{array}$$

where *a_ij_*, *a_ijkl_*, *a_ijklmn_*, and *a_ijklmnop_* are the dielectric stiffness coefficients measured under constant stress conditions. For BaTiO_3_, an 8th order expansion was used to describe the stability of the lattice polarization. The thermodynamic coefficients are adapted from ref. [59] to describe the effects of quantum fluctuations at cryogenic temperatures. ref. [60]

The intrinsic free energy density of the electronic polarization, in the absence of the lattice polarization, is described by

(12) $$f^{e}\;\left(T,\;P_{i}^{e}\right)=\frac{1}{2\varepsilon_{0}}\;B_{ij}^{e,ref}\left(T\right)P_{i}^{e}P_{j}^{e}$$

where $B_{ij}^{ref}\left(T\right)$ is related to the refractive index of the equivalent high symmetry phase. The coupling energy density between the lattice and electronic polarization, which determines the electro‐optic effect, is given by

(13) $$f^{L-e}\;\left(P_{i}^{L},\;P_{i}^{e}\right)=g_{ijkl}^{LL}\;P_{l}^{L}P_{k}^{L}P_{i}^{e}P_{j}^{e}$$

where $g_{ijkl}^{LL}$ relates the lattice polarization to the refractive index.

The gradient energy density is represented by

(14) $$f_{grad}=\frac{1}{2}\;G_{ijkl}\frac{\partial P_{i}^{L}}{\partial x_{j}}\frac{\partial P_{k}^{L}}{\partial x_{l}}$$

where *G_ijkl_* is the gradient energy tensor where the non‐zero coefficients are chosen to be *G*_11_ =  0.6,  *G*_22_ =   − 0.6, and *G*_44_ =  0.6, and the units are normalized by $\alpha_{1}l_{o}^{2}$ where α_1_ is the first Landau expansion coefficient and *l_o_* is chosen as 1 nm per grid.

The elastic energy is given by

(15) $$f^{elas}\;\left(P_{i}^{L},\;P_{i}^{e},\;\sigma_{ij}\right)=C_{ijkl}\;\left(\varepsilon_{ij}-\varepsilon_{ij}^{o}\right)$$

where *C_ijkl_* is the elastic stiffness, ε_*ij*_ is the total strain, and $\varepsilon_{ij}^{o}$= $Q_{ijkl}\sigma_{ij}P_{k}^{L}P_{l}^{L}+\frac{1}{2\varepsilon_{0}}\pi_{ijkl}\sigma_{ij}P_{k}^{e}P_{l}^{e}$ is the eigenstrain, where *Q_ijkl_* is the electrostrictive coefficient and π_*ijkl*_ is the piezo‐optic tensor for the high‐symmetry phase. The total strain was solved for a thin film boundary condition assuming that the strain relaxes to its equilibrium value at each time step; for simplicity the contribution of the electronic polarization to the eigenstrain was ignored. Further details on solving the elasticity may be found in ref. [61]

The electrostatic energy is given by

(16) $$f^{elec}=-E_{i}P_{i}^{L}-E_{i}P_{i}^{e}-\frac{1}{2}\varepsilon_{0}\kappa_{ij}^{b}E_{i}E_{j}$$

where ε_0_ is the vacuum permittivity and $\kappa_{ij}^{b}$ is the background dielectric constant. For simplicity, to compute the evolution of the lattice polarization, only the linear contribution to the electronic polarization was included and added to the background dielectric constant yielding $f^{elec}=-E_{i}P_{i}^{L}-\frac{1}{2}\varepsilon_{0}\kappa_{ij}^{b}E_{i}E_{j}$, where $\kappa_{ij}^{b}=10$ and is isotropic. Here $\kappa_{ij}^{b}$ contains contributions from the electronic contribution and the vacuum and other hard modes. Appendix A and Table S5 (Supporting Information) contain all coefficients used in the phase‐field simulations.

For the simulations, a system size of 128 Δ*x*_1_ × 128 Δ*x*_2_  × 56 Δ*x*_3_ was used. There were 12 Δ*x*_3_ grid points for the substrates where the elastic constants were assumed to be the same as the ferroelectric film, and there are 4 Δ*x*_3_ grid points acting as an air layer above the film. The thickness of BaTiO_3_ was set to be 40∆x_3_. Periodic boundary conditions were used along the in‐plane lateral directions, and natural boundary conditions were used along the film‐substrate and film‐air interfaces. The interface between the film and the substrate was assumed to be coherent, and hence the misfit strain was calculated using the equivalent cubic lattice parameters for BaTiO_3_ and the pseudocubic lattice parameters for GdScO_3_.^[^ ^62^ ^]^

(17) $$\varepsilon_{11}=\frac{a_{GdScO3}^{{\left[110\right]}_{o}}-a_{BaTiO3}^{eq}}{a_{BaTiO3}^{eq}},\varepsilon_{22}=\frac{a_{GdScO3}^{{\left[001\right]}_{o}}-a_{BaTiO3}^{eq}}{a_{BaTiO3}^{eq}},\;\varepsilon_{12}=\varepsilon_{21}=0$$

where the lattice parameters for BaTiO_3_ are $a_{BaTiO3}^{eq}=4.01Å\;\left(1+1.15\times 10^{-5}\left(T-300\;K\right)\right)$ and GdScO_3_ are $a_{GdScO3}^{{\left[110\right]}_{o}}=3.970Å\;\left(1+1.09\times 10^{-5}\left(T-300\;K\right)\right)$ and $a_{GdScO3}^{{\left[001\right]}_{o}}=3.966\;Å\;\left(1+1.09\times 10^{-5}\left(T-300\;K\right)\right)$.

The simulations begin with an initial condition of $P_{i}^{L}\;\left(x_{i},\;t=0\right)=\left[0.0\;0.0\;0.1\;C\;m^{-2}\right]+\Delta P^{noise}\left(x_{i},\;t=0\right)$, where |*P^noise^* |  =  0.1 C m^−2^. To simulate the evolution of the polarization under an applied electric field and the corresponding electro‐optic effect, a uniform electric field was applied along the [100] direction ranging from −70 to 70 kV cm^−1^. The electric field was incremented by 0.7 kV cm^−1^ every 100 timesteps. The linear electro‐optic effect and quadratic electro‐optic effect were found by taking the average of the refractive index over the ferroelectric material as a function of an applied electric field and fitting to a polynomial expansion centered around zero. The simulations for the evolution of the lattice polarization were completed using the mu‐Pro software.

### Synchrotron‐Based Reciprocal Space Mapping

Synchrotron X‐ray diffraction experiments were performed at the ID4B (QM2) beamline at the Cornell high energy synchrotron source (CHESS). The incident X‐ray energy was 20 keV. An area detector array (Pilatus 6M) was used to collect the scattering intensities in a grazing incidence reflection geometry (8° incidence angle). The sample was rotated through 360° rotations, sliced into 0.1° frames. Geometric parameters of the Pilatus 6M detector, such as detector distance, tilting, rotation, and direct beam position, were extracted using a standard CeO_2_ powder reference.

## Conflict of Interest

The authors have a provisional patent filed on this work. Long‐Qing Chen has a financial interest in MuPRO, LLC, a company which licenses and markets the software package used in this research.

## Supporting information



Supporting Information

## Data Availability

The data that supports the findings of this study are available within the article. Datasets used for the generation of figures presented in the article is available at https://doi.org/10.5281/zenodo.17296490. Additional data related to the film growth and structural characterization is available at https://doi.org/10.34863/k6vg‐fr77. Any additional data connected to the study are available from the corresponding author upon reasonable request.
