# Supplementary material for: Colossal Cryogenic Electro‐Optic Response Through Metastability in Strained BaTiO3 Thin Films
Source: Adv Mater. 2025 Oct 11;38(3):e07564. doi: 10.1002/adma.202507564 (PMC12801356; doi:10.1002/adma.202507564)
Supplement: Supplementary file 1 — Supporting Information [file ADMA-38-e07564-s001.docx]

**Supporting Information**

**Colossal Cryogenic Electro-Optic Response through Metastability in Strained BaTiO_3_ Thin Films**

Albert Suceava*, Sankalpa Hazra*, Aiden Ross*, Ian Reed Philippi, Dylan Sotir, Brynn Brower, Lei Ding, Yingxin Zhu, Zhiyu Zhang, Himirkanti Sarkar, Saugata Sarker, Yang Yang, Suchismita Sarker, Vladimir A. Stoica, Darrell G. Schlom, Long-Qing Chen and Venkatraman Gopalan

**Note S1: Structural Characterization of BaTiO_3_ on GdScO_3_ Thin Films**


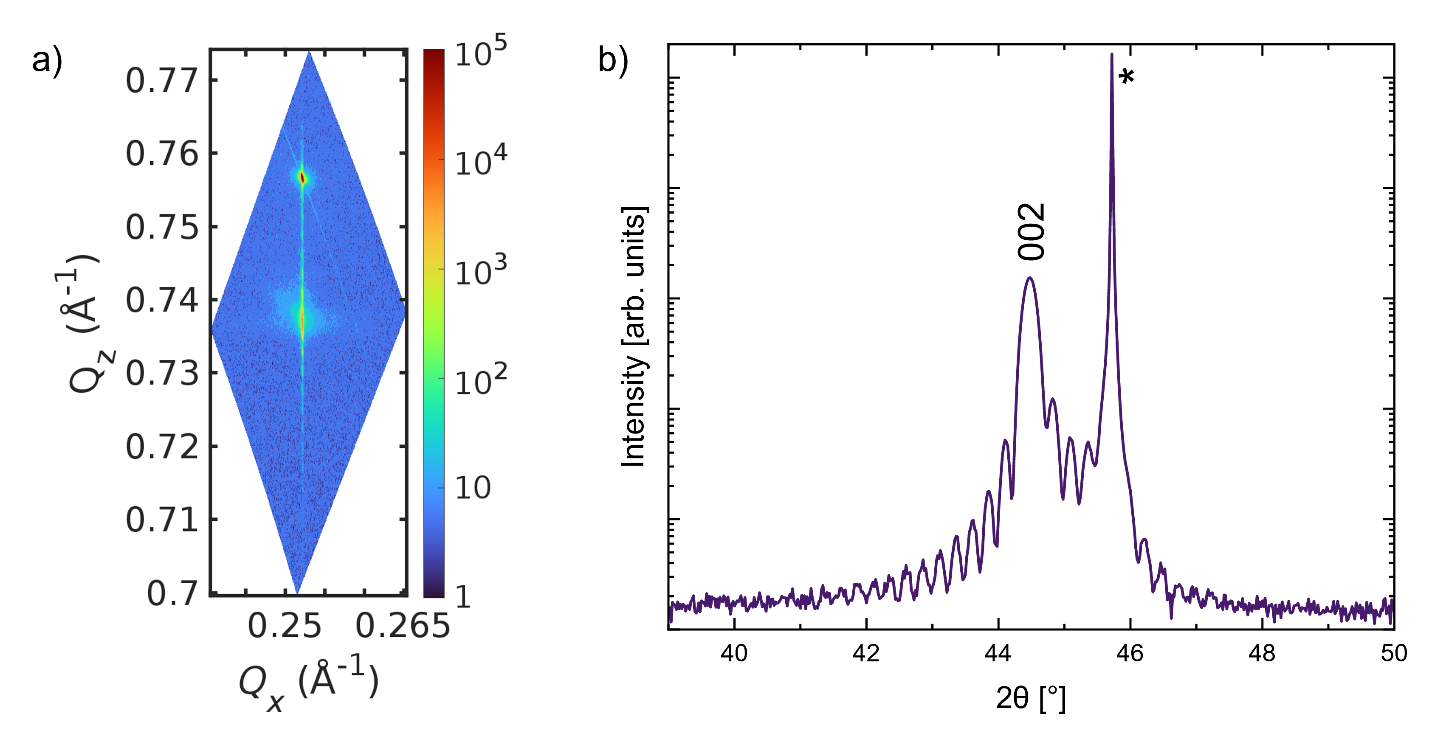


**Figure S1.** a) Asymmetric reciprocal space map scan taken at the GdScO_3_ 332 peak, demonstrating epitaxial growth. b) *θ-*2*θ* x-ray diffraction scan showing an approximately 37 nm thick film of BaTiO_3_ grown on GdScO_3_ (110)_o_ at *T_sub_* = 1160 °C at a background pressure of 10% ozone of 1×10^–6^ Torr. The *c*-axis lattice constant, measured from the RSM, is 4.073 ± 0.003 Å. The film is commensurately strained to the substrate and shows only *c*-axis orientation. This can be compared to the predicted *c*-axis lattice constant of ideal BaTiO_3_ commensurately strained to GdScO_3_ (110), which is calculated using the elastic stiffness tensor in Voigt notation and experimentally determined lattice parameters of BaTiO_3_ and the GdScO_3_ substrate (which has a congruently melting composition that differs from the stoichiometric composition):^[1–3]^

$$c_{\perp}=c_{BaTiO_{3}}+\frac{\left( {4a}_{BaTiO_{3}}-c_{GdScO_{3}}-\sqrt{a_{GdScO_{3}}^{2}+b_{GdScO_{3}}^{2}} \right){c_{BaTiO_{3}}c}_{13}}{2a_{BaTiO_{3}}c_{33}}$$

The predicted value is 4.077 Å, which is close to the out-of-plane lattice constant of this film. This is also in agreement with Matsubara *et al*. who demonstrated commensurately strained BaTiO_3_ films on GdScO_3_ (110) substrates, grown in an adsorption-controlled regime via metalorganic gas-source MBE, with a *c*-axis lattice parameter of 4.074 Å.^[4]^

**
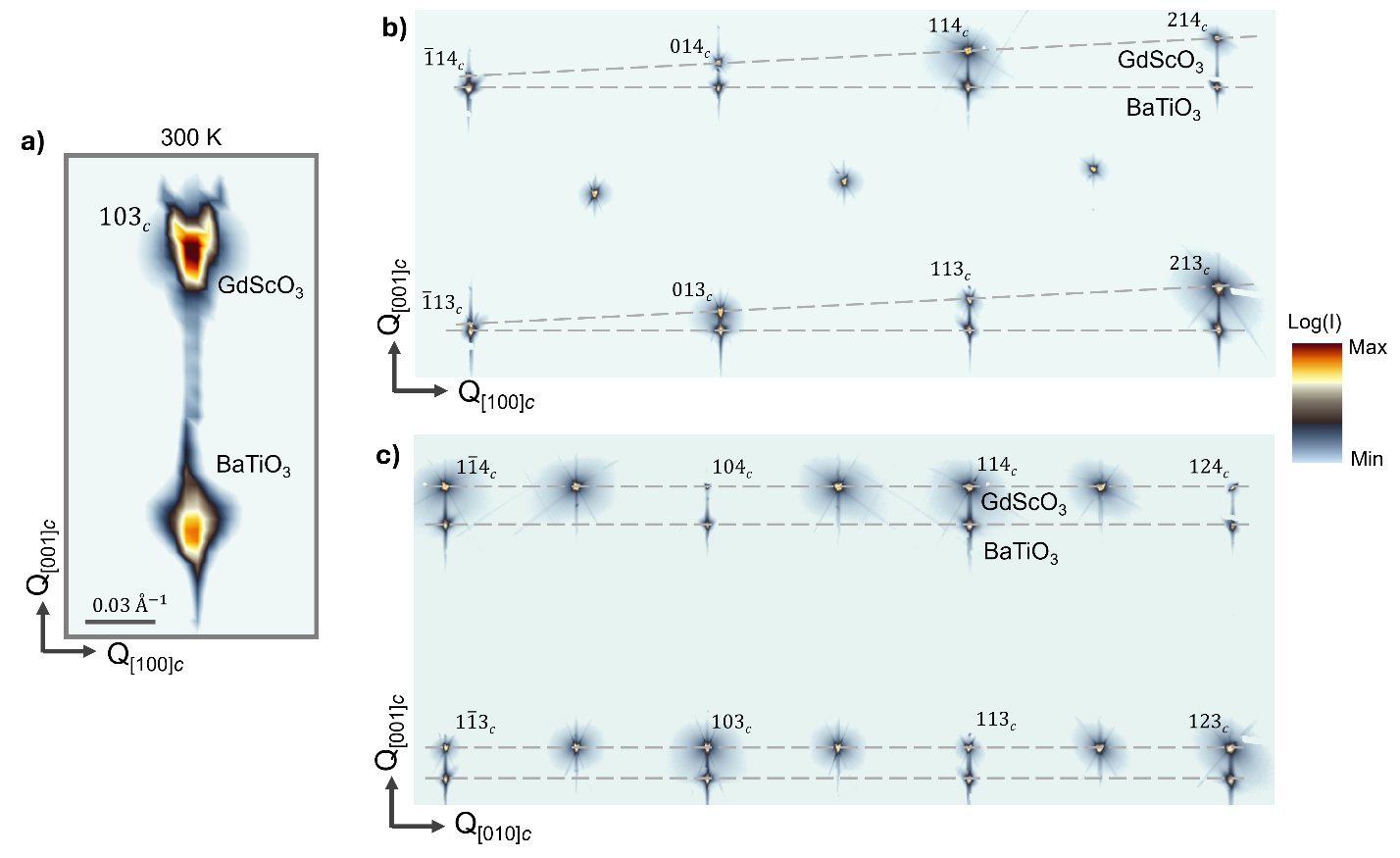
**

**Figure S2.** a) Room temperature X-ray reciprocal space maps (RSM) around the 103*_c_* GdScO_3_ peak (*c* stands for cubic notation) showing the epitaxially coherent nature of the BaTiO_3_ film. b) Large area reciprocal space planes (normal to [010]*_c_* direction) showing tilted series of GdScO_3_ peaks related to the orthorhombic nature of the substrate while the films peaks are aligned with the crystallographic direction as expected for a tetragonal structure of the BaTiO_3_ thin films at room temperature. (c) Similar large area RSMs normal to the [100]*_c_* direction displaying no such tilts in either GdScO_3_ substrate peaks or BaTiO_3_ thin film peaks.

**
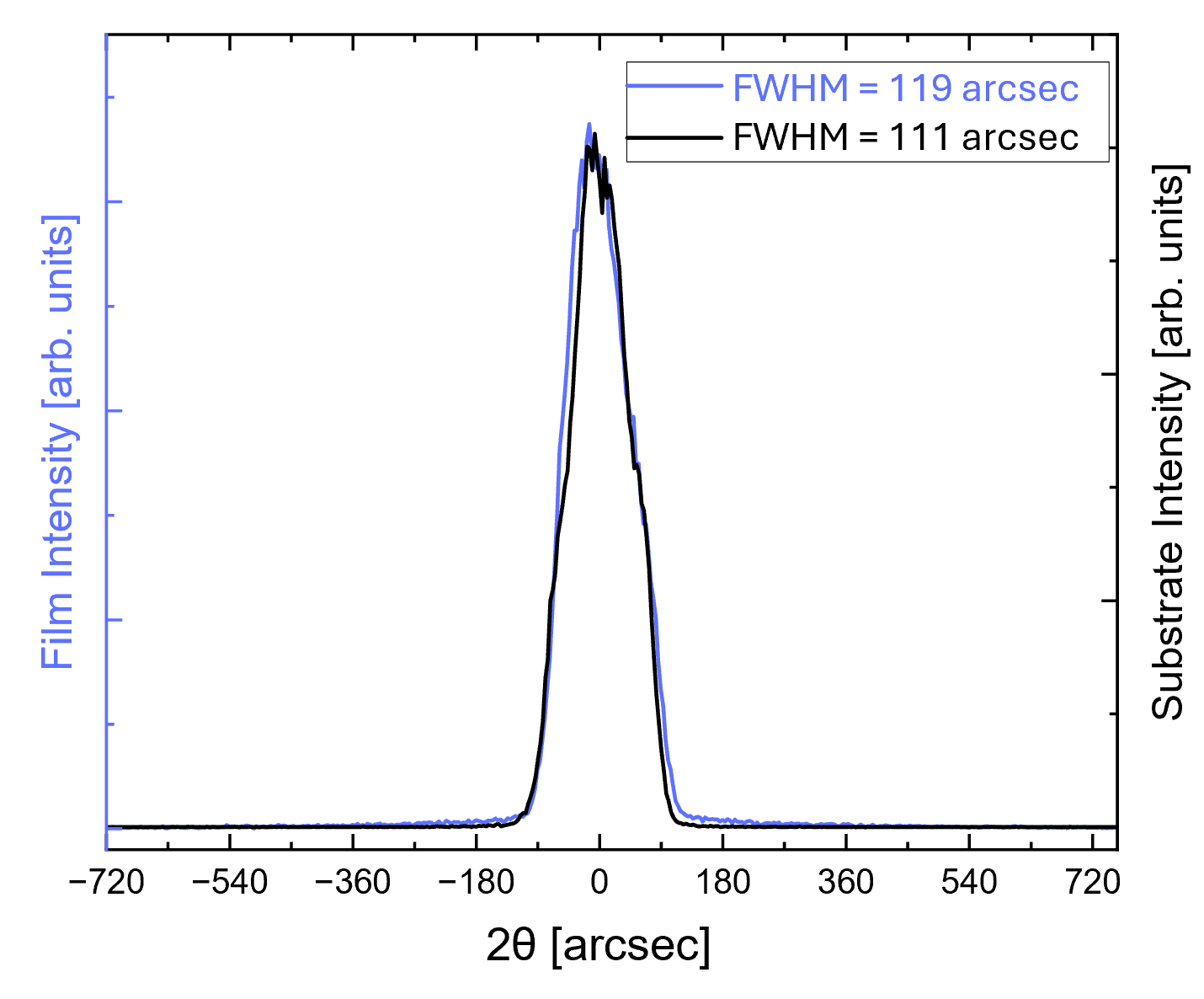
**

**Figure S3.** Rocking curve measurement comparing the film and the substrate, taken at the BaTiO_3_ 002 and the GdScO_3_ 220 diffraction peaks.

**
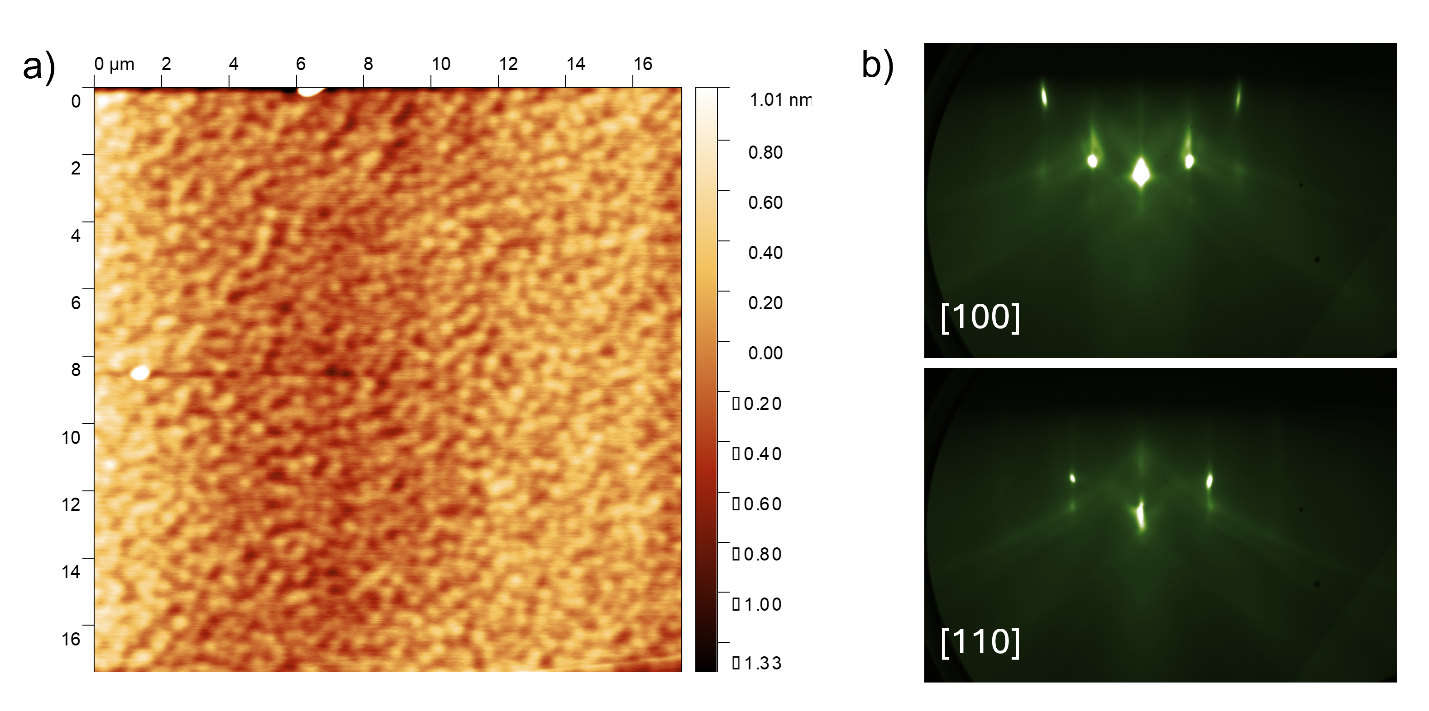
**

**Figure S4.** a) Atomic force microscope scan of the film surface, with a root-mean-square roughness of 392 pm. b) RHEED images of the film post growth, taken at room temperature along the azimuths of BaTiO_3_ indicated.

**
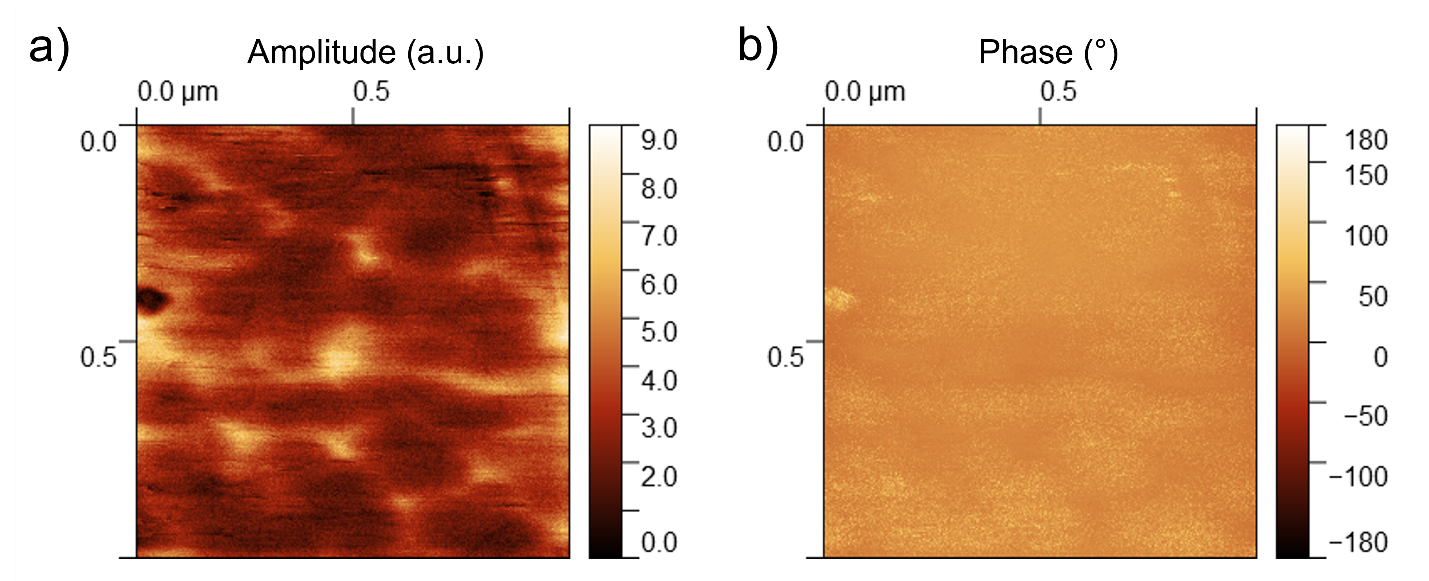
**

**Figure S5.** a) Amplitude and b) phase signal channels of room temperature piezoelectric force microscopy scans collected from a 1 $\times$ 1 μm region of the film. No contrast in phase is apparent, suggesting the absence of antipolar tetragonal domains.


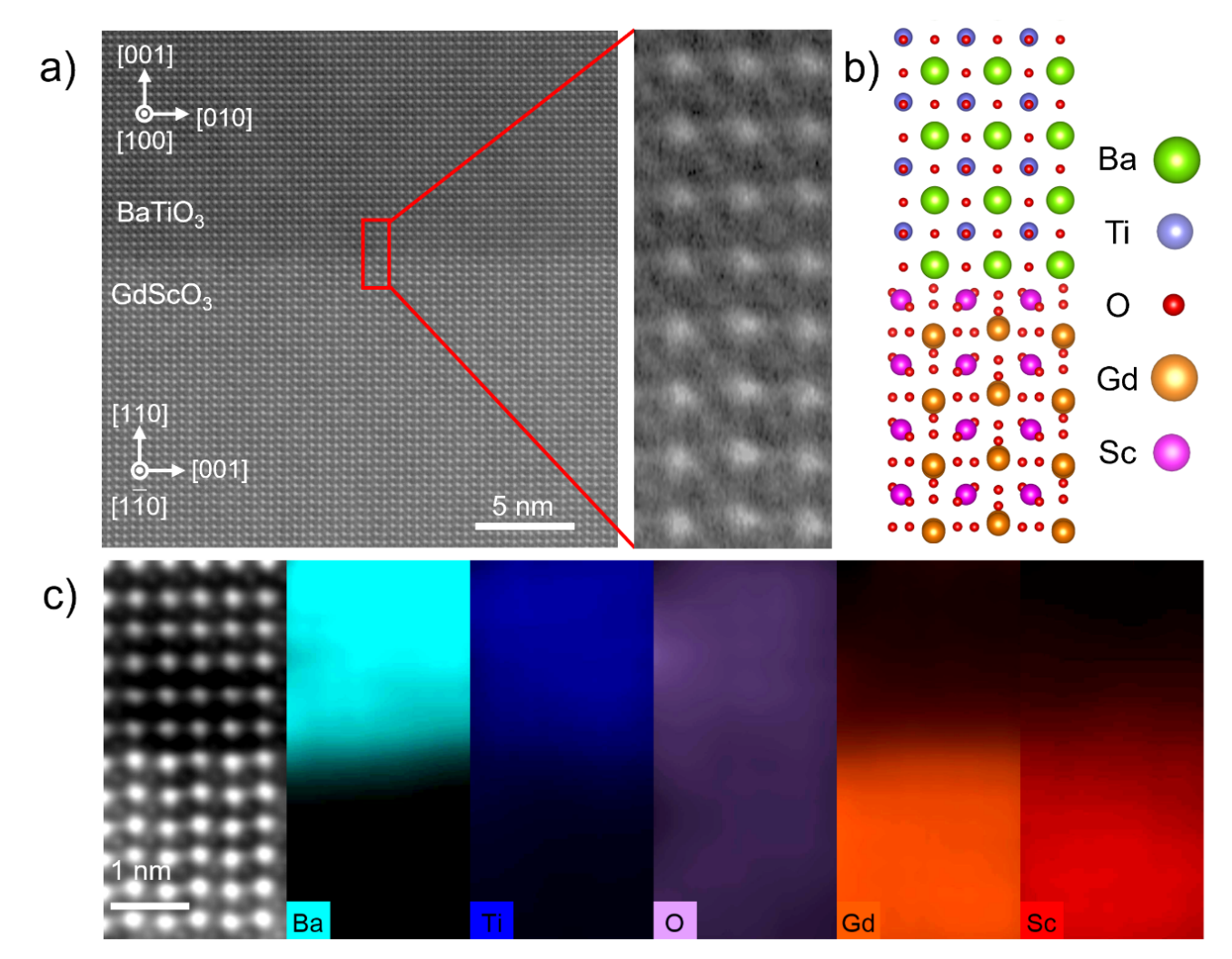


**Figure S6.** a) HRSTEM image of BaTiO_3_ and GdScO_3_ interface, demonstrating coherent growth. b) Schematic of interfacial structure. c) Energy-dispersive X-ray Spectroscopy images displaying the mass percent distribution maps of elemental species at the interface.


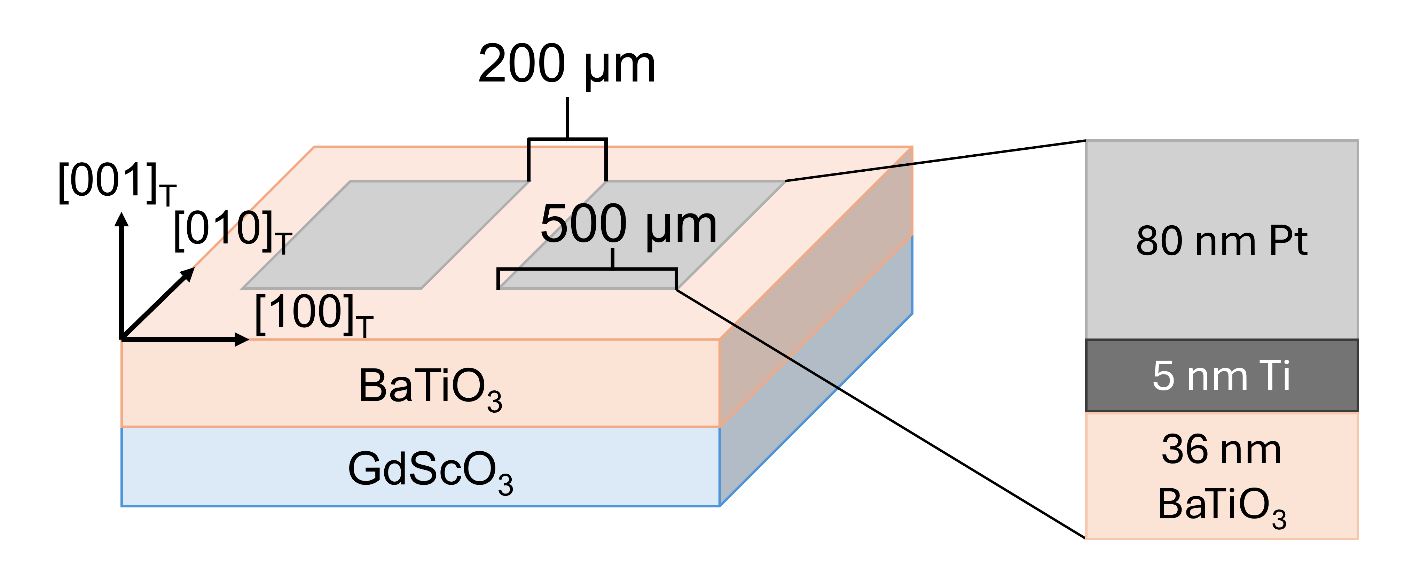


**Figure S7.** Schematic of surface deposited square electrodes for applying an in-plane electric field.


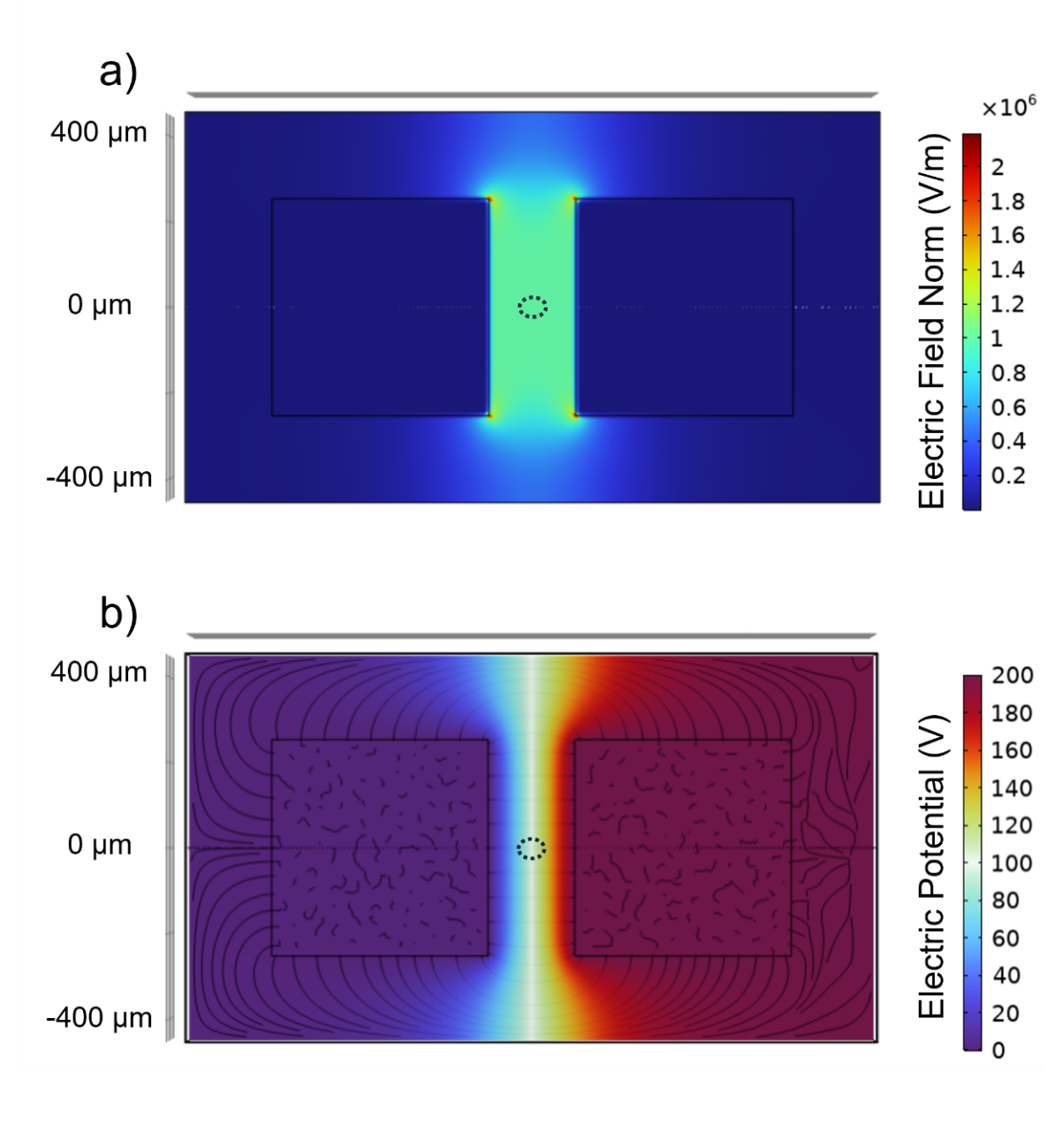


**Figure S8.** a) Electric field magnitude and (b) electric potential between surface deposited electrodes as simulated in the COMSOL Multiphysics Electrostatics module. Vector field streamlines have been added to the electric potential visualization as a guide to the eye. The simulation environment consists of a 37 nm film layer on top of a 200 nm substrate layer. Their dielectric constants are 2000 and 20 respectively ^[5, 6]^. Two 500 μm square surfaces are defined on the film surface, with one set to ground and the other a 200 V potential. The COMSOL calculated electric field value of 10 kV/cm in the electrode gap matches the expected value obtained when dividing the voltage applied by the electrode separation. The dotted ellipses indicate the relative full width half max diameter of the focused beam used for electro-optic characterization, projected onto the film surface at 45° incidence.


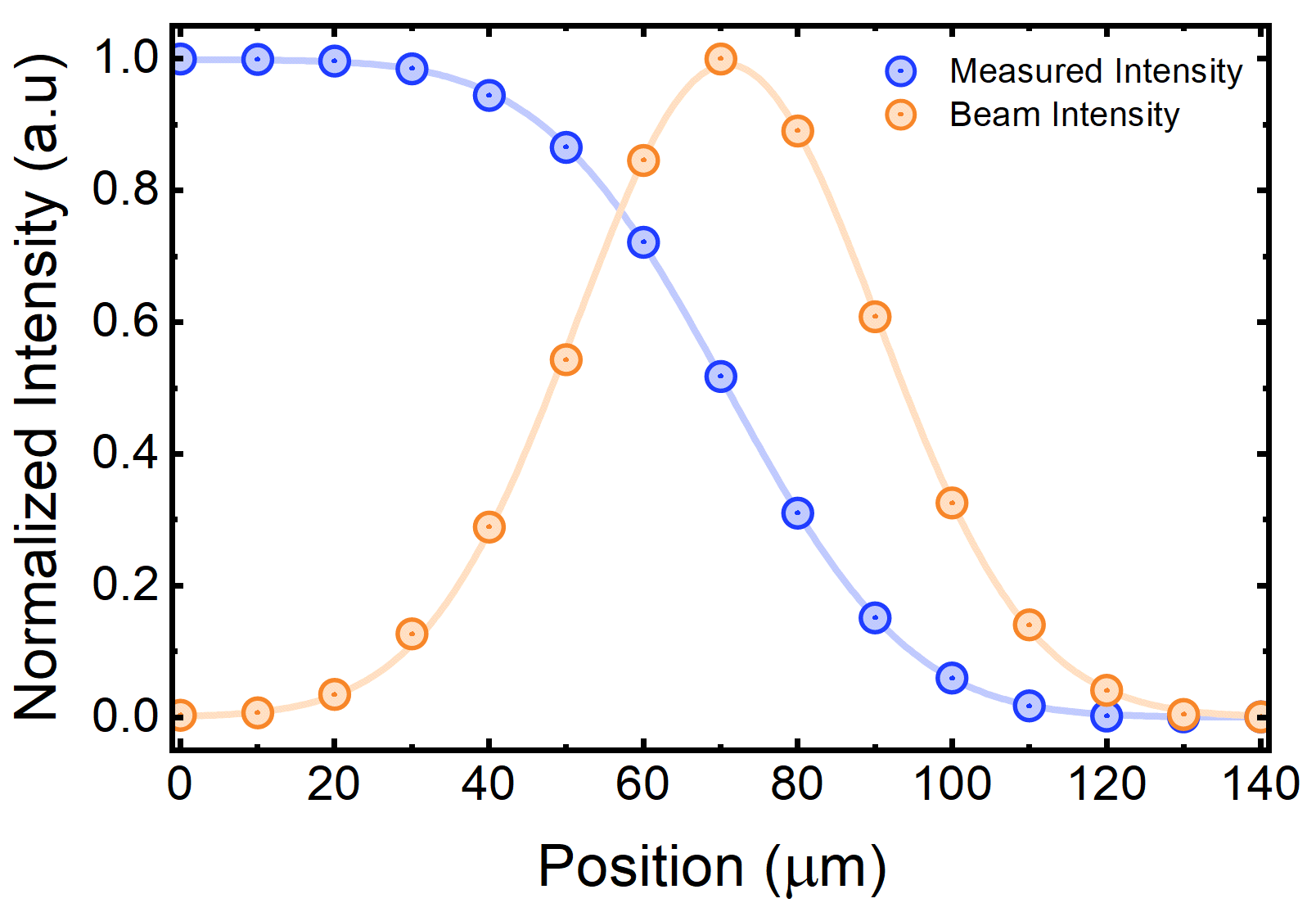


**Figure S9.** Knife edge measurements of 1550 nm probe profile utilized for electro-optic measurement. The experimentally measured signal strength is shown with blue data points, while the gradient of the experimental data reflecting the probe intensity profile is shown with orange data points. Solid lines indicate fits to data of the form $I=I_{0}\cdot\frac{1}{2}\left( 1-\mathrm{erf} \left[ \frac{x-x_{0}}{\sigma\sqrt{2}} \right] \right)$ and $\frac{dI}{dx}=-I_{0}\frac{\exp\left[ -\left( x-x_{0} \right)^{2}/2\sigma^{2} \right]}{\sigma\sqrt{2\pi}}$ respectively. The full width half max beam diameter is 43.9 μm and the diameter measured from the ${1/e}^{2}$ level is 74.6 μm.

**Note S2: Validation of Electro-Optic Measurement Against a Standard Sample**

In order to develop confidence in the function of the PSCA setup and the analysis of experimental data, measurements were performed on a reference 10x10x1 mm X-cut LiNbO_3_ single crystal obtained from MTI Corporation. Electrodes were prepared such that a field could be applied along the [0001] crystal axis, corresponding to the crystal physics 3 direction, by blanket sputtering 100 nm of Pt on both faces of the crystal while using a strip of Kapton tape to shield a thin region in the center of each face. This preparation resulted in a 1.85 mm electrode gap and electrical continuity between sputtered Pt on both faces, minimizing fringing effects and maximizing the homogeneity of the 3-oriented field within the electrode gap.

Measurement was performed at normal incidence with an electric field applied along the crystal physics 3 direction, resulting in the birefringence of the crystal being modulated according to the $r_{13}$ and $r_{33}$ coefficients. Since $r_{13}$ and $r_{33}$ will work in unison to modulate the sample birefringence, neither coefficient can be measured in isolation; an effective response $r_{eff}=r_{33}-\left( \frac{n_{o}^{3}}{n_{e}^{3}} \right)r_{13}$ is observed. An effective electro-optic coefficient of $r_{eff}=23.4$ pm/V can be retrieved from the slope of the linear electro-optic response shown in **Figure S9b**. This is to be compared against a predicted value of $r_{eff}=21.3$ pm/V based on material property parameters provided by the supplier, demonstrating a reasonable level of agreement.


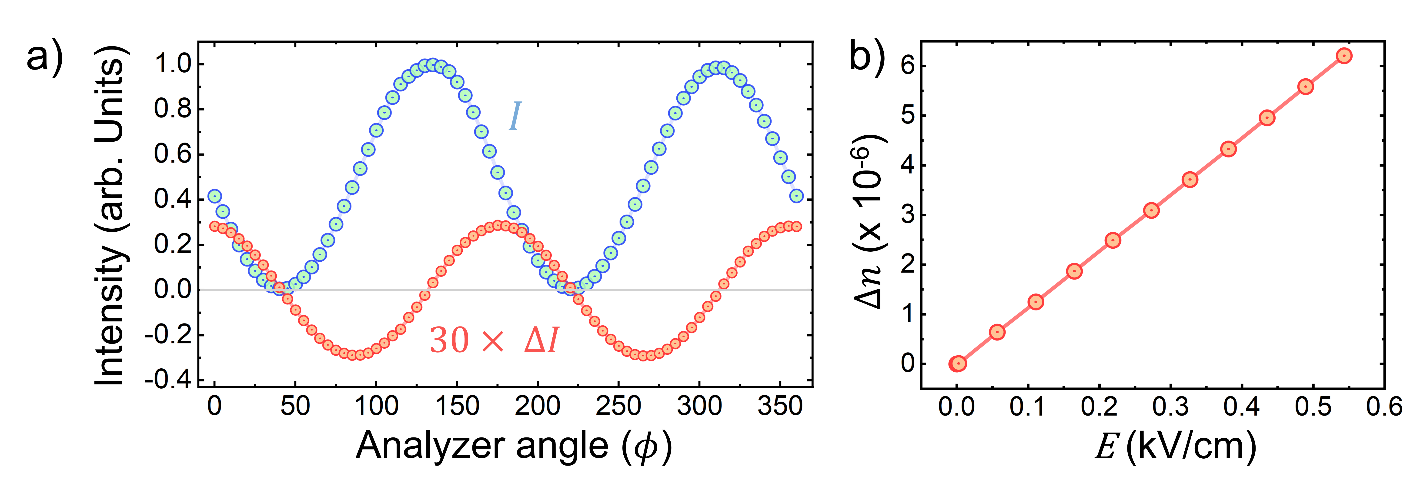


**Figure S10.** a) Modulated and unmodulated intensity curves retrieved from measurement of an X-cut LiNbO_3_ single crystal. (b) The observed refractive index change as a function of electric field applied to the single crystal, demonstrating a clear linear response.

**Note S3: Linear Optical Properties of Strained BaTiO_3_ Film**

To quantify the room temperature refractive index of the sample, spectroscopic ellipsometry measurements were performing using a J. A. Woollam M-2000XI rotating-compensator, variable angle spectroscopic ellipsometer, with Ψ, Δ, and Mueller matrix parameters collected at incident angles of 45°, 55°, and 65°. Data was collected across the 271.1 to 1689.3 nm spectral range and fitting was performed within the J.A. Woollam CompleteEASE software. The complex refractive index values $n$ and $k$ are extracted from Ψ, Δ, and $M_{ij}$ spectra using a least-squares regression analysis and unweighted error function to fit constructed dielectric function models of a semi-infinite GdScO_3_ substrate and BaTiO_3_ film layers to experimental data.

An anisotropic model of the film refractive index accounting for sample birefringence was developed as follows. Firstly, measurements were performed on a sample of the GdScO_3_ substrate and a dielectric model following the Cauchy equation: $n\left( \lambda\right)=A+\frac{B}{\lambda^{2}}+\frac{C}{\lambda^{4}}$ , where $\lambda$ is in units of μm. An Urbach absorption tail was included in the model to account for a non-zero imaginary component of the refractive index, *k*, of the form: $k=k_{amp} e^{G(E-E_{g})}$. We note that in the Urbach tail equation, $k_{amp}$ and $E_{g}$ are coupled variables during fitting, and so $E_{g}$ does not represent a physical parameter with a unique solution. Fit parameters obtained for the GdScO_3_ substrate are: $A=2.001$, $B=0.01125$, $C=0.00029005, G=0.443, E_{g}=0.443$.

Secondly, a model is constructed for fitting data collected on a sample of the BaTiO_3_, film studied grown on GdScO_3_, with the GdScO_3_ dielectric function developed in previous fitting step used with the fit parameters fixed. An isotropic model was constructed from the data set collected at 45°, assuming that the sample index observed reflects $n_{o}$ nearly completely ($n_{e}$ corresponding to light polarized along the out-of-plane polar direction of the tetragonal unit cell). A dielectric function model consisting of multiple oscillator functions is then constructed. Two separate models, one consisting of Lorentz oscillators and the other consisting of Gaussian oscillators, were developed and considered. The functional form of the Lorentz oscillators is given by ${\varepsilon_{Lorentz}}_{i}=\frac{{Amp}_{i}\cdot{Br}_{i}\cdot{En}_{i}}{{En}_{i}^{2}-E^{2}-i\cdot E\cdot{Br}_{i}}$, with fit parameters ${Amp}_{i}$, ${Br}_{i},$ and ${En}_{i}$ representing the amplitude, width, and center position of the $i^{th}$ oscillator respectively, and $E$ the photon energy expressed in eV. The form of the Gaussian oscillators is given by ${\varepsilon_{Gaussian}}_{i}={Amp}_{i}\left\{ \left[ \Gamma\left( \frac{E-{En}_{i}}{\sigma_{i}} \right)+\Gamma\left( \frac{E+{En}_{i}}{\sigma_{i}} \right) \right]+i\cdot\left( \exp\left[ -\left( \frac{E-{En}_{i}}{\sigma_{i}} \right)^{2} \right]-\exp\left[ -\left( \frac{E+{En}_{i}}{\sigma_{i}} \right)^{2} \right] \right) \right\}$, where $\sigma_{i}=\frac{{Br}_{i}}{2\sqrt{\ln(2)}}$ and Γ is a convergence series that produces a Kramers-Kronig consistent line shape for $\varepsilon_{1}$.^[7–9]^ The primary difference between the two oscillators lies in the peak shape in the imaginary part of the dielectric function corresponding to the resonance of the oscillator. The Gaussian oscillator provides a much steeper tail of the oscillator away from the center energy, resulting in sharper band edges and reduced extinction coefficients away from the band edge. The real part of the film dielectric function is further affected by a wavelength-independent constant, $\varepsilon_{\infty}$, and UV and IR poles following Lorentz oscillators with zero-broadening that reflect resonances far outside the measurement spectral range: $\varepsilon_{IR}=\frac{{Amp}_{IR}}{{En}_{IR}^{2}-E^{2}}$ and $\varepsilon_{UV}=\frac{{Amp}_{UV}}{{En}_{UV}^{2}-E^{2}}$. An initial guess for the film thickness of $t=37$ nm was used, based on XRR measurements performed for initial structural characterization.

After the fit parameters ${Amp}_{i}$, ${Br}_{i},$ and ${En}_{i}$ are allowed to relax against the 45° data set, further fitting is performed against the 65° data set. The BaTiO_3_ layer model is made anisotropic, with the extraordinary axis oriented in the out-of-plane direction and the extraordinary index determined by summing the ordinary optical constants with “difference” values calculated from an extended Cauchy dispersion equation: ${d\varepsilon}_{i}$.^[7]^ The extended Cauchy equation resembles that used to fit the optical constants of the GdScO_3_ substrate, with the inclusion of a higher order term and IR pole. The difference parameters serve as fitting parameters for the extraordinary index and are allowed to relax to fit the 65° incidence data set, with the ordinary optical constants fixed.

Following the initial generation of the extraordinary index, both the ordinary and extraordinary optical constants are allowed to relax to simultaneously fit all angles in the data set: 45°, 55°, and 65°. Once the optical fit parameters are relaxed against the complete data set, the optical constants are fixed and the film thickness made a new fit parameter to be relaxed until a minimum in mean-squared error is reached. Alternating relaxation of the fits for film optical constants and film thickness is performed until a global minimum in all parameters is reached. The final models developed for the film layer are described below in **Table S1** and the accompanying text:

**Table S1: Fit parameters for optical constants of BaTiO_3_ film at room temperature**

|  | Lorentz | | | Gaussian | | |
| --- | --- | --- | --- | --- | --- | --- |
| Oscillator Number | ${Amp}_{i}$ | ${Br}_{i}$ | ${En}_{i}$ | ${Amp}_{i}$ | ${Br}_{i}$ | ${En}_{i}$ |
| 1 | 0.324128 | 5.0491 | 1.828 | 0.668082 | 5.0302 | 1.873 |
| 2 | 4.259748 | 0.1964 | 3.781 | 7.428887 | 0.2808 | 3.756 |
| 3 | 4.377518 | 0.4444 | 4.118 | 5.664778 | 0.3357 | 4.112 |
| 4 | 7.828694 | 0.6844 | 4.705 | 8.632928 | 0.6736 | 4.677 |

Additional fit parameters include:

For the Lorentz oscillator model: $t=36.53$ nm, $\varepsilon_{\infty}=0$, ${Amp}_{IR}=0.2831$, ${En}_{IR}=0 eV$, ${Amp}_{UV}=438.5576$, ${En}_{UV}=11.125 eV$, ${d\varepsilon}_{A}=-0.335015$, ${d\varepsilon}_{B}=-0.402018$, ${d\varepsilon}_{C}=0.080858$, ${d\varepsilon}_{D}=-0.004421$, ${d\varepsilon}_{IR}=0.207897$.

For the Gaussian oscillator model: $t=36.53$ nm, $\varepsilon_{\infty}=0$, ${Amp}_{IR}=0.2831$, ${En}_{IR}=0 eV$, ${Amp}_{UV}=438.5576$, ${En}_{UV}=11.125 eV$, ${d\varepsilon}_{A}=-0.029490$, ${d\varepsilon}_{B}=0.974320$, ${d\varepsilon}_{C}=-0.060582$, ${d\varepsilon}_{D}=0.002233$, ${d\varepsilon}_{IR}=-0.050425$.

For determination of the cryogenic refractive index, a modified Oxford Instruments MicrostatHe cryostat was integrated with the aforementioned Woollam M-2000XI. The cryostat windows were constructed to be normal to an incident probe at 65°, resulting only in modulation of the spectroscopic ellipsometry probe intensity without affecting measured Ψ, Δ and $M_{ij}$ quantities when data was collected at that angle. As such, Ψ, Δ and $M_{ij}$ spectra were collected for an incident angle of 65° and at 5 K. During the cooling process, the cryostat chamber was pumped to a vacuum of 10^-5^ at room temperature and no sample icing was observed, which would lead to periodic fluctuations in Ψ and Δ with wavelength if at least one the order of a hundred nanometers in thickness. Thus, all changes in Ψ, Δ and $M_{ij}$ measured at 5 K are attributed to changes in the refractive index of the BaTiO_3_ film. The existing room temperature birefringent model was allowed to relax in order to best fit the low temperature data. While a monoclinic structure is expected to be biaxial, the limited geometry within the cryostat reduces the ability to sample the anisotropy of the sample. Furthermore, with 4 domain variants expected and a large probe spot size of 3x5 mm, the observed optical response is expected to sample all domain variants, with the in-plane index an aggregate of $n_{1}$ and $n_{2}$ and therefore effectively uniaxial. Oscillator fit parameters were fit sequentially, with each parameter initially constrained to within 10% of the room temperature value, and the constraints increasingly expanded until a minimum goodness of fit was achieved. The low temperature fit parameters for the film are given in **Table S2** below. The full collection of raw Ψ, Δ data and model fits for room temperature and low temperature experiments are provided in **Figure S11** below.

**Table S2: Fit parameters for optical constants of BaTiO_3_ film at 5 K**

|  | Lorentz | | | Gaussian | | |
| --- | --- | --- | --- | --- | --- | --- |
| Oscillator Number | ${Amp}_{i}$ | ${Br}_{i}$ | ${En}_{i}$ | ${Amp}_{i}$ | ${Br}_{i}$ | ${En}_{i}$ |
| 1 | 0.623944 | 3.2068 | 2.001 | 1.733072 | 4.6560 | 3.333 |
| 2 | 6.696964 | 0.9849 | 3.608 | 5.422329 | 0.8694 | 3.589 |
| 3 | 1.693879 | 0.2187 | 4.119 | 1.906398 | 0.2528 | 4.131 |
| 4 | 3.216761 | 0.6612 | 4.627 | 3.182036 | 0.5070 | 4.581 |

Additional fit parameters include:

For the Lorentz oscillator model: $t=36.53$ nm, $\varepsilon_{\infty}=0.708$, ${Amp}_{IR}=0.3066$, ${En}_{IR}=0 eV$, ${Amp}_{UV}=210.3801$, ${En}_{UV}=11.792 eV$, ${d\varepsilon}_{A}=-0.441993$, ${d\varepsilon}_{B}=-0.423672$, ${d\varepsilon}_{C}=0.078737$, ${d\varepsilon}_{D}=-0.004368$, ${d\varepsilon}_{IR}=0.265581$.

For the Gaussian oscillator model: $t=36.53$ nm, $\varepsilon_{\infty}=0.698$, ${Amp}_{IR}=0.2730$, ${En}_{IR}=0 eV$, ${Amp}_{UV}=210.3801$, ${En}_{UV}=11.792 eV$, ${d\varepsilon}_{A}=-0.100343$, ${d\varepsilon}_{B}=0.323656$, ${d\varepsilon}_{C}=-0.053430$, ${d\varepsilon}_{D}=0.00089873$, ${d\varepsilon}_{IR}=0.219749$.


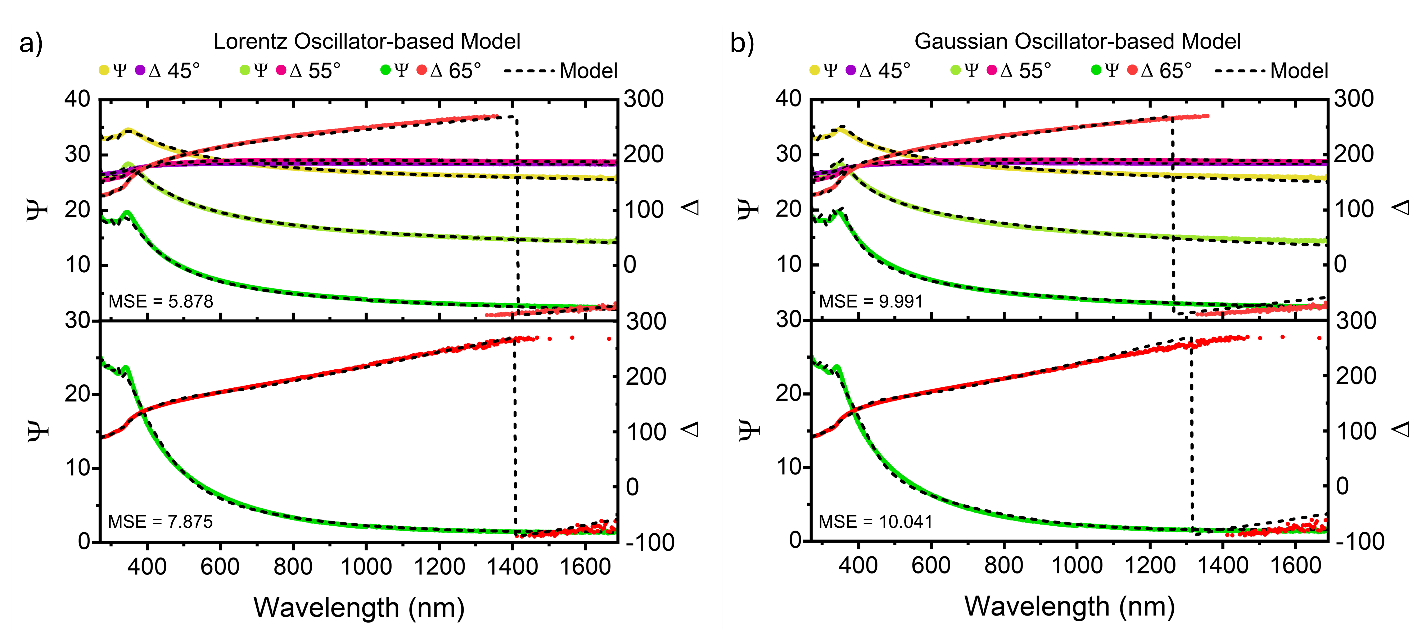


**Figure S11.** Raw Ψ, Δ experimental data collected at room temperature (top) and 5 K (bottom) and resulting fits to data generated using a (a) Lorentz oscillator-based model and (b) Gaussian oscillator-based model as described in Supplemental Note 3. Mean squared error (MSE) values demonstrating the goodness of fit of the models are provided within each plot.

In order to supplement the spectroscopic ellipsometry measurements, an Agilent - Cary 5000 system was used to collect normal incidence UV-Vis spectroscopy specular transmittance measurements on the film that was the focus of the study and similar samples, with the results presented below in **Figure S12**. In addition, a free space near-normal incidence reflectance of the BaTiO_3_ thin film at 1550 nm was also measured at room temperature. The backside of the substrate was polished at an angle, leading to a wedge geometry that isolates the reflection from only the air-thin film and thin film-substrate interfaces. Collected at a 4° angle of incidence, an absolute normalized reflected intensity of 0.124 was measured. Using a thin film reflection model accounting for multiple reflections and sample birefringence, a predicted value of 0.1237 is obtained using optical constants retrieved from the Gaussian oscillator-based spectroscopic ellipsometry model, indicating excellent agreement.^[10]^ In contrast, the Lorentz oscillator-based model predicts a reflectance of 0.1292, yielding agreement only within 4%. As a consequence of these results, the optical constants provided by Gaussian oscillator-based model are used for evaluation of electro-optic coefficients derived within the main text, as shown in **Figure 2(e-f)**. The dispersion curves obtained by using the Lorentz oscillator-based model are provided below in **Figure S13**.


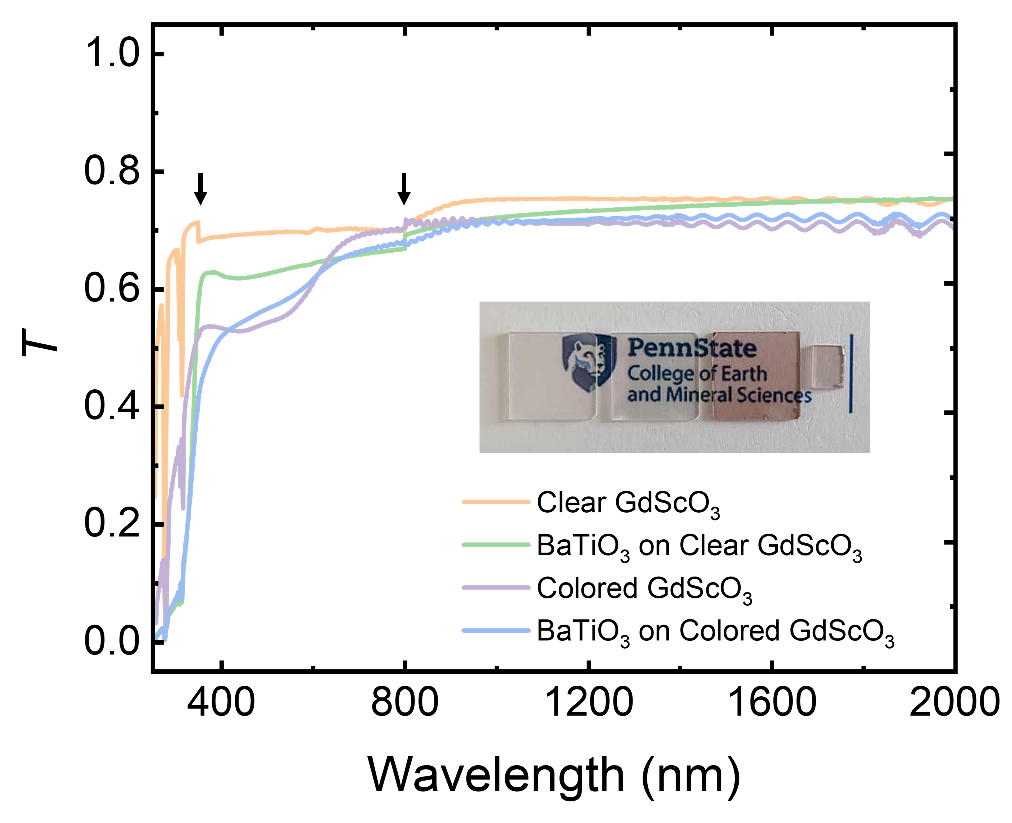


**Figure S12.** Normal incidence transmittance spectra obtained via performing UV-Vis spectroscopy on BaTiO_3_ on GdScO_3_ film characterized in main text (deposited on colored substrate) along with transmittance spectra collected from a similarly grown film on a visibly transparent GdScO_3_ substrate, a transparent GdScO_3_ substrate, and a colored GdScO_3_ substrate. Variations in the transparency of GdScO_3_ substrates can occur due to excess Gd, leading to an orange tint and the reduced transmittance observed in the spectra of the film deposited on a colored substrate and a colored substrate. The discontinuities in the spectra indicated by arrows near 350 nm and 800 nm are due to a change in the light source and detector respectively. (inset) A picture taken showing the optical transparency of the set of samples. From left to right: clear GdScO_3_, BaTiO_3_ film on clear GdScO_3_, colored GdScO_3_, BaTiO_3_ film on colored GdScO_3_. Use of the Penn State mark is granted for this one-time editorial use with no promotional use granted.


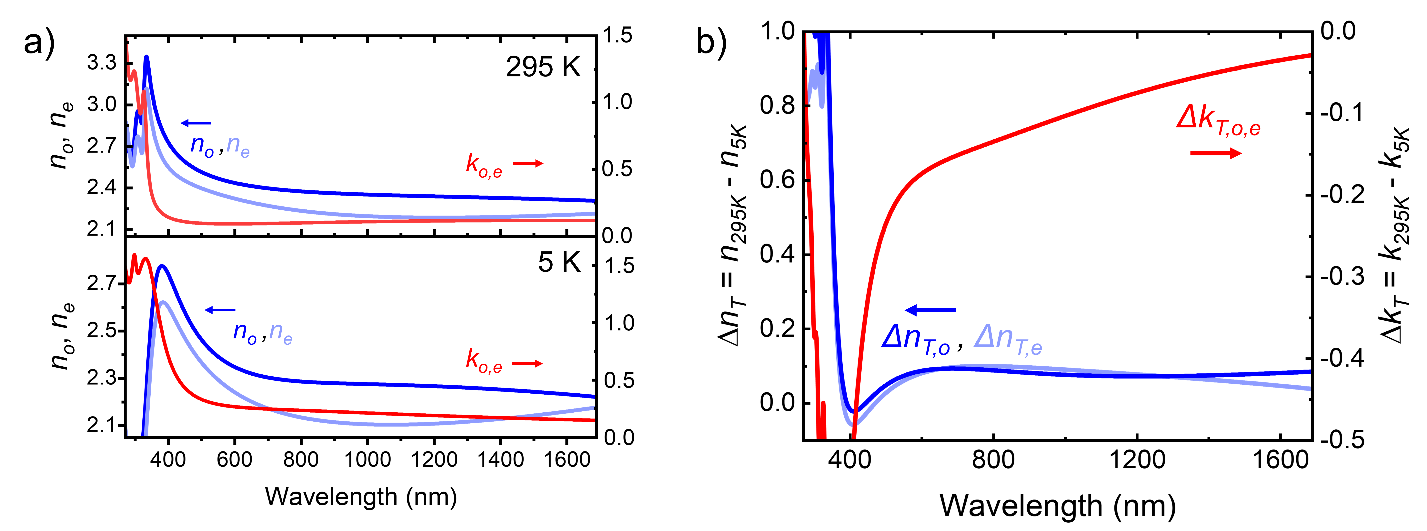


**Figure S13.** a) Refractive index and extinction coefficient of strained BaTiO_3_ film versus wavelength, at room temperature and at 5 K, as determined by variable angle ellipsometry using a Lorentz oscillator-based model as compared to the Gaussian oscillator-based model presented in **Figure 2(e-f)**. b) Relative refractive index and extinction coefficient changes between room temperature and 5 K, $\Delta n_{T}=n_{295K}-n_{5K}$ , $\Delta k=k_{295K}-k_{5K}$.


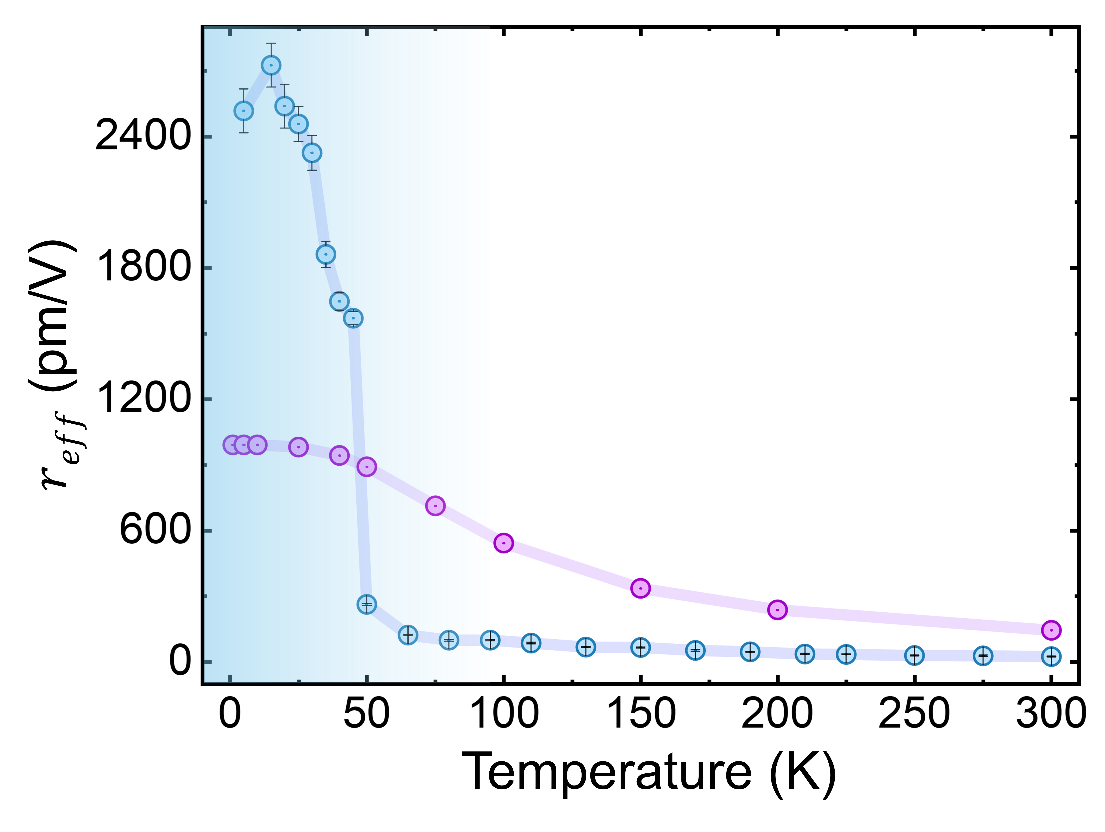


**Figure S14.** Temperature dependence of maximum electro-optic response, derived using a linear interpolated of refractive index values between the experimentally measured values at 295 K and 5 K.

**Note S4: Commentary on the Tensor Nature of the Electro-Optic Response and Behavior of the Index Ellipsoid**

In order to optimize the sample geometry for measurement, the tensor nature of the electro-optic response must be diligently considered. The tetragonal BaTiO_3_ films that are the focus of this work were grown with the [001] direction out of plane so that an in-plane applied electric field would be along the [100] direction, corresponding to the crystal physics direction 3 and 1 respectively. Consequently, the refractive index of the film is described through the perturbed index ellipsoid:^[11]^

$\frac{x^{2}}{n_{1}^{2}}+\frac{y^{2}}{n_{2}^{2}}+\frac{z^{2}}{n_{3}^{2}}+2xzr_{51}E_{1}=1$. (S1)

Note that the cross section obtained by setting $z=0$ will remain the equation of a circle for a uniaxial material with $n_{1}=n_{2}$ regardless of the magnitude of $E_{1}$. This means that for a beam propagating along $3$, such that the set of polarization states spans the plane spanned by $1$ and $2$ and the range of corresponding refractive indices experienced by such waves is described by the circle $\frac{x^{2}}{n_{o}^{2}}+\frac{y^{2}}{n_{o}^{2}}=1$, an applied electric field will not induce an observable refractive index change. For this reason, the film is rotated by 45° in the path of the probe as illustrated by **Figure 2a.**

**
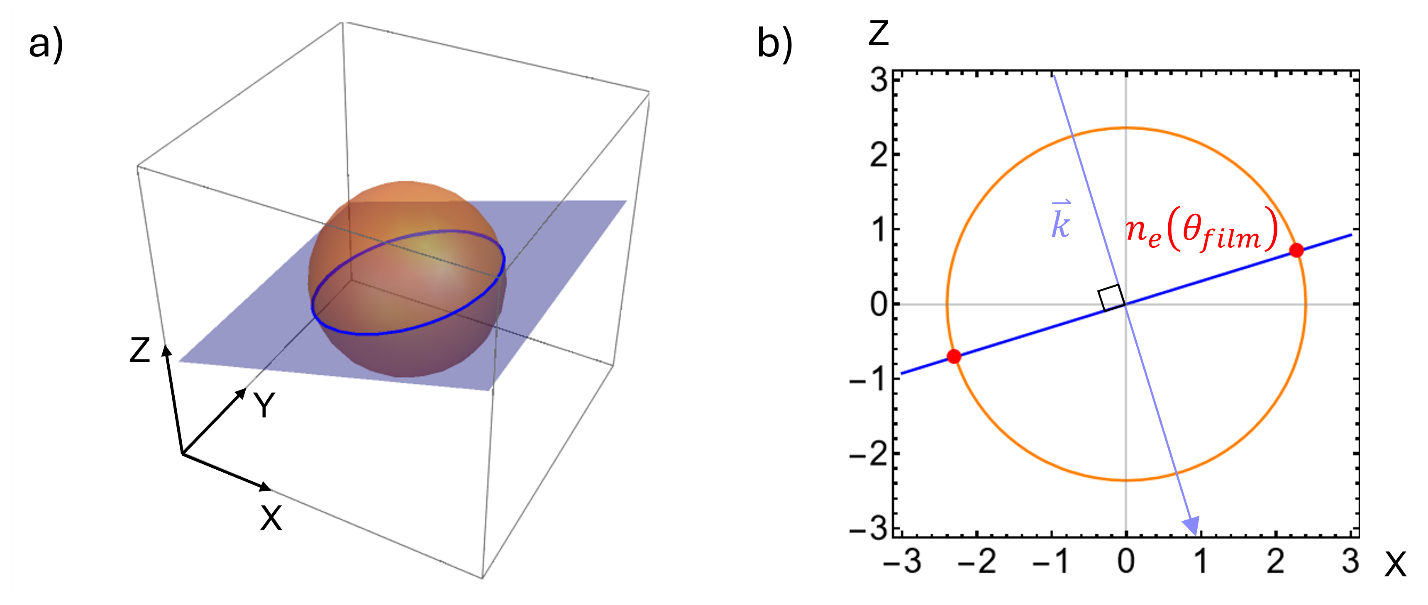
**

**Figure S15.** a) The intersection of the index ellipsoid described by Equation S1 with $E=0$ and the plane described by Equation S2(c). The ellipse that defines the intersection carries a semi-minor axis of $n_{o}$ and a semi-major axis of $n_{e}(\theta_{film})$. b) The same intersection viewed in the $y=0$ plane, used to calculate $n_{e}(\theta_{film})$ as indicated by the red points. A relationship between $n_{e}(\theta_{film})$ and an applied field $E$ can be used to convert an observed index change to a specific tensor quantity.

Successful interpretation of the electro-optic response relies upon deliberate consideration of the tensor nature of the property, especially as the symmetry of the structure is reduced and more tensor elements contribute to the experimentally measured response. The electro-optic response in the tetragonal phase was previously discussed in main text Section 3.1 and above. To briefly reiterate, the sample geometry was such that only $r_{51}$ contributes to the observed refractive index change. The action of $r_{51}$ results in a nonzero dielectric tensor element in the unperturbed crystal physics coordinate frame, effectively rotating the crystal physics axis about the 2 axis while “stretching” the index ellipsoid in the 1-3 plane. In order to convert the measured $r_{eff}$ to the tensor element $r_{51}$, we geometrically solve for a fixed point on the surface of the perturbed index ellipsoid in accordance with the following prescription.

Consider the ellipsoid in the unperturbed crystal physics axis coordinate system, following **Equation 2** with $E=0$. The *z*-axis coincides with the 3 direction and the out-of-plane direction of the sample. An incident probe at 45° propagates through the film at a reduced angle, $\theta_{film}$ according to Snell’s law. For simplicity, the splitting of the beam into extraordinary and ordinary rays is not considered, only the propagating angle for the ordinary ray which is roughly 17° from the normal for the unperturbed ellipsoid. With the propagation direction of the probe through the film defined by this angle, one can formulate the equation of a plane describing the span of polarization states for this ray:

$p=\left\{ \cos\theta_{film},0,\sin\theta_{film} \right\} s=\left\{ 0,1,0 \right\}$ (S2a)

$n=p\times s$ (S2b)

$n_{x}x + n_{y}y +n_{z}z=0$ (S2c)

The intersection of **Equation S2c** with the index ellipsoid of **Equation 2** yields the cross-sectional ellipse whose perimeter describes the refractive indices experienced by the propagating ray. Since perturbation of the ellipsoid with a field along 1 will leave the 2-axis undisturbed (2 = 2′, $n_{2}=n_{2}^{'}=n_{o}$), we set $y=0$ to view the cross-sectional perimeter in the *xz* plane and the plane of **Equation S2c** as a line. The intersection of these two reveals $n_{e}(\theta_{film})$. The experimentally measured $\Delta n=n_{e}\left( \theta_{film} \right)'-n_{e}(\theta_{film})$, where $n_{e}\left( \theta_{film} \right)'$ is obtained by following the same procedure in the same coordinate system but using the equation for the perturbed ellipsoid under an applied field with $E\neq0$. With this approach, one can work backwards to relate the measured $\Delta n$ to an exact value of $r_{51}$, as shown in **Figure S12**.

For the monoclinic phase, the general approach remains valid. The crystal physics coordinate system remains unchanged through the phase transformation; the only additional consideration comes from the additional tensor elements present for the monoclinic point group *m*. For an electric field applied along 1, and the monoclinic mirror plane perpendicular to 2, the relevant additional components are the $r_{11}$ and $r_{31}$ coefficients. If we consider a full 4-domain monoclinic structure as suggested by SHG fitting models, then for some domain variants the applied electric field will be along the 2 direction, resulting in contributions from $r_{42}$ and $r_{62}$. For such low symmetry structures, it becomes difficult then to isolate the contribution from a single tensor element.

In order to gauge the response from such other coefficients, EO measurements below 50 K were performed at normal incidence. **Such measurements yielded no detectable response**. Based on the noise floor of the experimental instrumentation, the smallest detectable electro-optic response for a film with the thickness and refractive index of the strained BaTiO_3_ would correspond to a $r_{eff}$ of 5 pm/V. Utilizing the 4-domain monoclinic model suggested by SHG analysis, we can expect two set of domain twins, referred to as *D*_1_ (monoclinic mirror plane perpendicular to tetragonal [100] = 1) and *D*_2_ (monoclinic mirror plane perpendicular to tetragonal [010 = 2]). Expressed in the reference frame of the crystal physics axis of the tetragonal system, their respective electro-optic property tensors are:

$D_{1}: \left( \begin{matrix} 0 & r_{112} & r_{113} \\ 0 & r_{222} & r_{223} \\ 0 & r_{332} & r_{333} \\ 0 & r_{232} & r_{233} \\ r_{131} & 0 & 0 \\ r_{121} & 0 & 0 \end{matrix} \right)$ $D_{2}: \left( \begin{matrix} r_{111} & 0 & r_{113} \\ r_{221} & 0 & r_{223} \\ r_{331} & 0 & r_{333} \\ 0 & r_{232} & 0 \\ r_{131} & 0 & r_{133} \\ 0 & r_{122} & 0 \end{matrix} \right)$

Thus, the coefficients that would contribute to a response for a field applied along 1 include $r_{111}=r_{11}$, $r_{221}=r_{21}$, $r_{331}=r_{31}$, $r_{131}=r_{51}$, and $r_{121}=r_{61}$. Under the normal incidence probe with a beam propagating along 3, the effects of $r_{31}$ and $r_{51}$ will not be observed, leaving $r_{11}$, $r_{21}$, and $r_{61}$. The inability to observe an electro-optic response under normal incidence suggests that either the magnitude of all these coefficients in the monoclinic phase is below our 5 pm/V limit, or their magnitudes are comparable such that even though the refractive indices along the 1 or 2 directions change, the birefringence of the material does not. With this in mind we assume that their effects do not contribute to the oblique incidence $r_{eff}$ observed in the monoclinic phase. This leaves only the $r_{31}$ and $r_{51}$ components as possible contributors to the monoclinic response at oblique incidence. From phase-field simulations, the predicted ratio between $r_{51}$ and $r_{31}$ reaches a minimum value of 170:1 at 1 K, as shown in **Figure S16**. Given the large discrepancy in their predicted magnitude, we elect to not consider the contribution from $r_{31}$. Thus, we attribute the measured $r_{eff}$ of the monoclinic response entirely to $r_{51}$, as shown in **Figure 1c** in the main text.

**
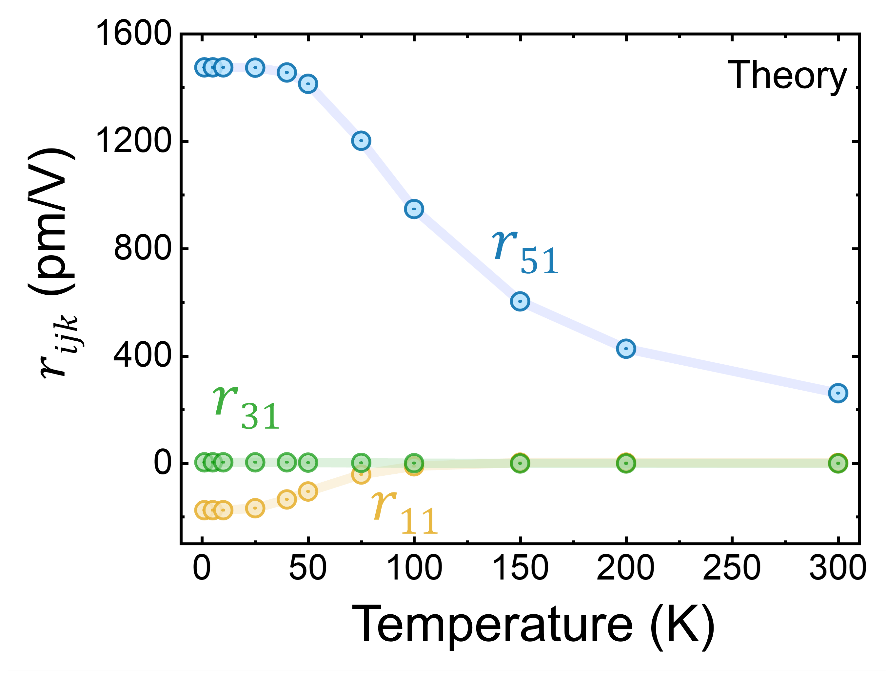
**

**Figure S16.** Individual electro-optic tensor elements as a function of temperature as predicted by phase-field simulations.

**Note S5: Consistency of Nonlinear Electro-Optic Response with Thermal and Electrical Cycling**


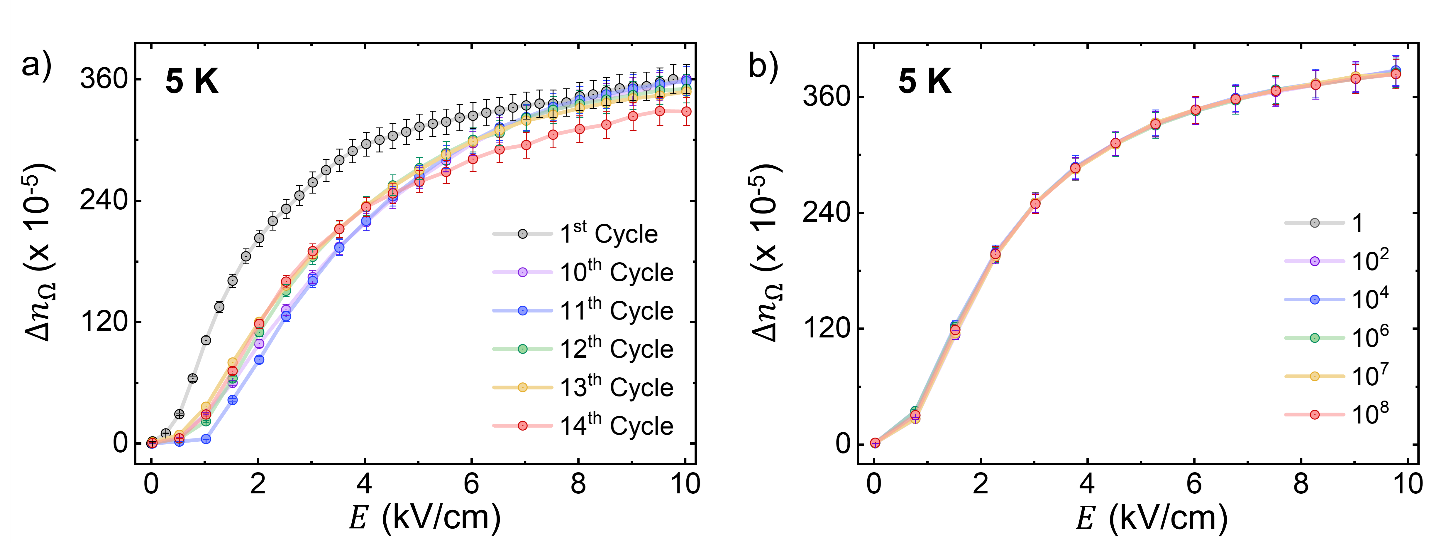


**Figure S17.** a) The enhanced nonlinear electro-optic response observed at 5 K across six different sample cooling runs, indicated with curves of different colors. b) The nonlinear electro-optic response recorded after subjecting the sample to increasing numbers of cycling under a 5 kV/cm, 20 kHz electric field.

To investigate the reproducibility of the electro-optic response, $\Delta n$ vs $E$ curves were collected and compared across multiple cooling-heating cycles and as a function of electrical cycling, as shown in **Figure S17**. The nonlinearity of the material response was observed to change after every instance the sample was cooled, suggesting a dependence on the initial low-temperature domain structure. The initial cooling cycle of the sample, which yielded the electro-optic data included in **Figures 1, 2,** and **3** of the main text is markedly different from those collected in later experiments. While the speed at which the change in refractive index is reached grew longer with subsequent cycles, the saturated value for the refractive index change remained consistent. These changes can be captured by fitting $r_{(1),max}$, $r_{(1),hf}$, and $r_{(1),lf}$, as described in the main text, with the results shown in **Table S3** below. We note that a reduced density of data points collected in the low field regime in the later cycles resulted in larger uncertainty in the coefficients retrieved from that regime.

**Table S3: Electro-optic coefficients extracted across multiple cooling cycles**

|  | $r_{(1),max}$ (pm/V) | $r_{(1),hf}$ (pm/V) | $r_{(1),lf}$ (pm/V) |
| --- | --- | --- | --- |
| Thermal Cycle 1 | $2516\pm90$ | $132\pm4$ | $475\pm79$ |
| Thermal Cycle 10 | $1252\pm22$ | $224\pm7$ | $70\pm6$ |
| Thermal Cycle 11 | $1460\pm44$ | $210\pm16$ | $152\pm100$ |
| Thermal Cycle 12 | $1591\pm35$ | $172\pm13$ | $93\pm22$ |
| Thermal Cycle 13 | $1516\pm36$ | $173\pm10$ | $182\pm49$ |
| Thermal Cycle 14 | $1619\pm36$ | $205\pm19$ | $63\pm26$ |

In comparison to the variation in material response observed with thermal cycling through the ~50 K phase transition, electrical cycling appears to have minimal effect on the nonlinearity of the sample response up to the number of high voltage cycles the sample was subjected to (10^8^). That is to say, once the system has been cooled below a phase transition the electro-optic response is highly robust and reproducible, but events that allow for the reconfiguration of the low-temperature domains can induce fluctuations in the sample response.


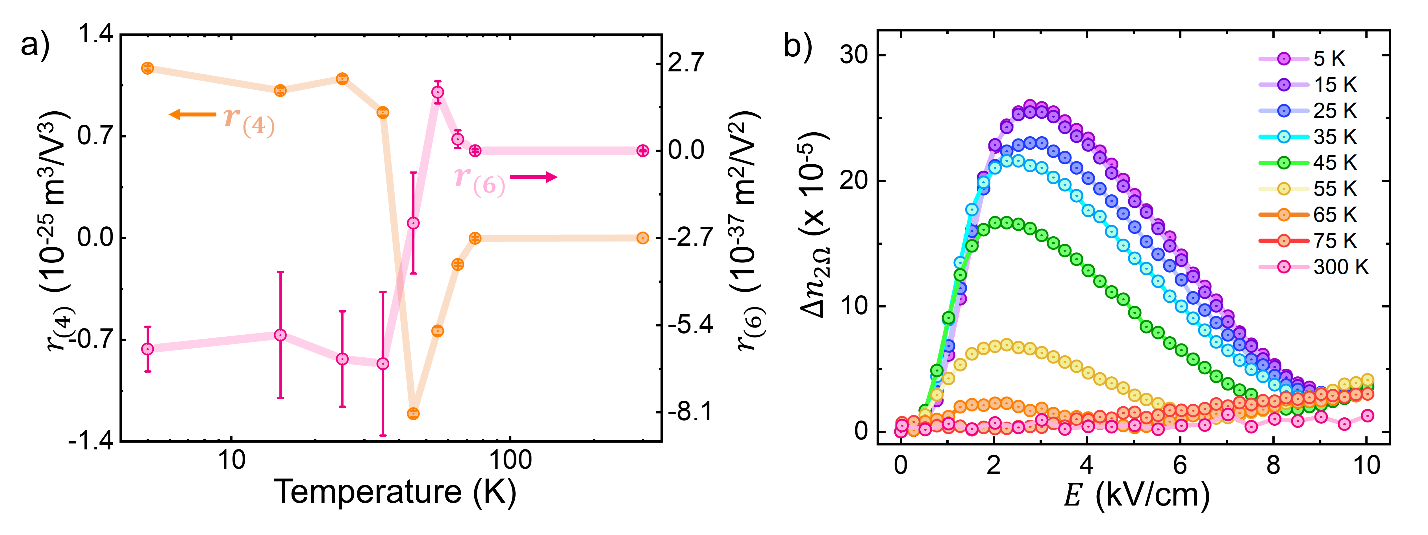


**Figure S18. a**) Fourth ($r_{4}$) and sixth ($r_{6}$) order electro-optic coefficients extracted from the low-field regime of the electro-optic response recorded at double the modulation frequency, as discussed in Section 3.2 and shown in **Figure 3c**. b) The temperature dependence of the second harmonic electro-optic response, containing even-order higher order electro-optic coefficients.

**Note S6: Phenomenological Model for Fitting the Nonlinear Electro-Optic Response**

To capture all three regimes of the nonlinear electro-optic response observed at the modulation frequency with a single equation, an approximate phenomenological model based on the Avrami-like model for the monoclinic phase is proposed:^[12]^

$\Delta n=A\left( 1-\exp\left( kE^{m} \right) \right)+BE$. (4)

The Avrami model is applicable to understanding solid-state transformation kinetics, where the fraction of a transformed phase is expressed as a function of time. The constants $k$ and $m$ are time-independent constants, where $m=D+1$ reveals the dimensions of growth, $D$, along which the transformation proceeds. In order to capture the full behavior of the S-curve, we add a leading constant $A$ to describe the overall magnitude of the index change and a linear term $BE_{amp}$ to describe the linear response in the saturation regime. We constrain $m$ to be 1 or 2 depending on whether the low-field quadratic regime is present or not (roughly below or above 35 K), leaving $k$ as an independent parameter related to the slope about the inflection point. In the low-field limit, this equation reduces to $\Delta n\sim AkE^{2}+BE$ when $m=2$ and $\Delta n=\left( Ak+B \right)E$ when $m=1$, allowing for the low-field quadratic behavior to be captured if present. In the high-field limit, the equation reduces to $\Delta n=A+BE$. Representative fits of the response at 5 K and 40 K are shown in **Figure S16**.


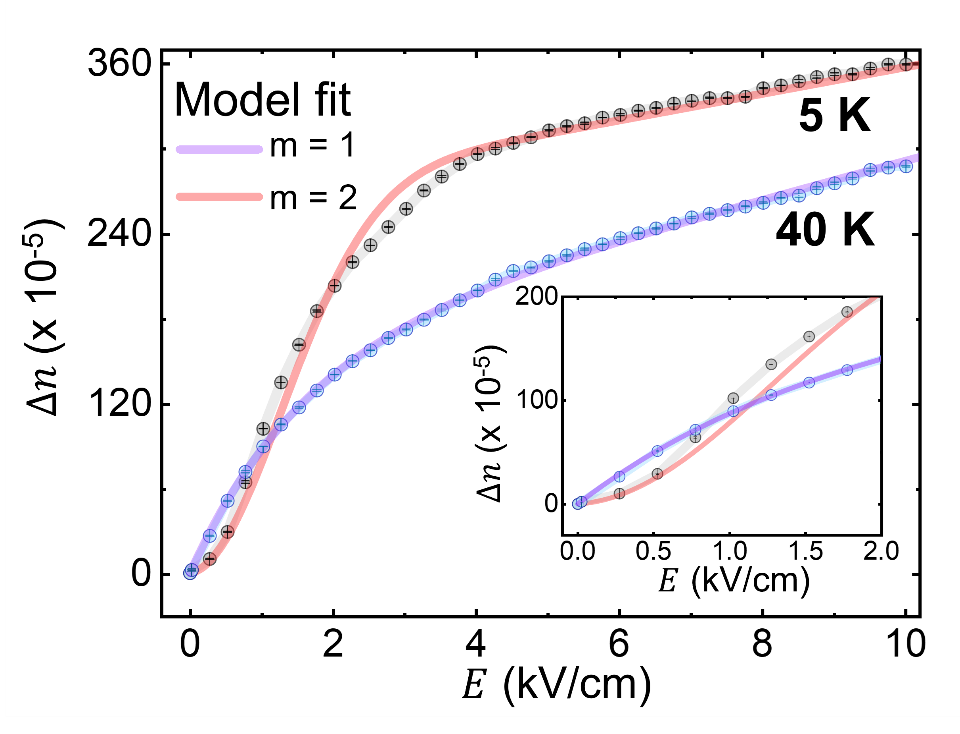


**Figure S19.** Phenomenological model fit of nonlinear response for two representative temperatures, demonstrating the emergence of low-field quadratic behavior.

**Note S7: Second-Harmonic Generation Polarimetry**

Due to similarities in crystal structure and symmetry, the model used to fit SHG polarimetry data follows in the framework developed in Ref. [13].^[13]^ For convenience, much of the information is reproduced below at the author’s permission:

A schematic of the SHG setup is shown in Supplemental **Figure S17**. The lab coordinates (X, Y, Z) are attached to the direction of the incoming beam: X || *p* polarization, Y || *s* polarization, where *p* and *s* polarization states are set by a rotating half wave plate. The crystal axes coordinate *i =* (1,2,3) are attached to the sample and related to the substrate as follows:

For GdScO_3_ substrates: 1 || [001]_o_ , 2 || [1-10]_o_ , 3 || [110]_o_ , where subscript “o” refers to orthorhombic unit cell of the scandate substrates.

In lab coordinates (X, Y, Z), the electric field of the incident beam can be written as $(E_{o}\cos\varphi, E_{o}sin \varphi, 0)$ where *φ* is the polarization rotation angle introduced by the half wave plate as shown in **Figure S20**. For an incidence angle θ on the sample, the electric field in the crystal axes coordinates can be expressed as $\left( E_{o}\cos\varphi\cos\theta,E_{o}\sin\varphi,-E_{o}\cos\varphi\sin\varphi) \right.$. The induced nonlinear polarization *P^2ω^*, is related to the incident electric field through the nonlinear susceptibility tensor, *d_ijk_* through the following equation:

$P_{i}^{2\omega}\propto d_{ijk}E_{j}^{\omega}E_{k}^{\omega}$ (1)

The proportionality constants depend on incident beam fluence, Fresnel’s coefficients at the film-air and film-substrate interfaces and the thickness of the films.

**Tetragonal model**:

For the tetragonal phase of point group 4*mm*, the nonlinear susceptibility can be written in Voigt notation as:

$$d_{ij}=\left( \begin{matrix} 0 & 0 & 0 & 0 & d_{15} & 0 \\ 0 & 0 & 0 & d_{15} & 0 & 0 \\ d_{31} & d_{31} & d_{33} & 0 & 0 & 0 \end{matrix} \right)$$

The induced nonlinear polarization in the crystal coordinates $i=\left( 1,2,3 \right)$ (calculated through equation 1) can be rotated back to lab coordinates (X, Y, Z), to give the *p* and *s*-polarized components of the SHG (*p* || X and *s* || Y). The *p* and *s*-polarized SHG intensities can be expressed as follows:

For normal incidence ($\theta=0^{\circ}$)

$I_{p}^{2\omega}\propto\left( P_{p}^{2\omega} \right)^{2}=0$

$$I_{s}^{2\omega}\propto\left( P_{s}^{2\omega} \right)^{2}=0$$

For oblique incidence $(\theta=45^{\circ})$

$I_{p}^{2\omega}\propto\left( P_{p}^{2\omega} \right)^{2}\propto\left( (2d_{15}-d_{31}-d_{33})cos[\varphi]^{2}-2d_{31}sin[\varphi]^{2} \right)^{2}$ (2)

$$I_{s}^{2\omega}\propto\left( P_{s}^{2\omega} \right)^{2}\propto d_{15}^{2}Sin[2\varphi]^{2}$$

**Monoclinic model**:

For the low temperature monoclinic phase with point group *m*_,_ a multi-domain model is assumed. In crystal physics coordinates $i=\left( 1,2,3 \right)$, nonlinear susceptibility in the monoclinic phase can be written as:

$$d_{ij}=\left( \begin{matrix} d_{11} & d_{12} & d_{13} & 0 & d_{15} & 0 \\ 0 & 0 & 0 & d_{24} & 0 & d_{26} \\ d_{31} & d_{32} & d_{33} & 0 & d_{35} & 0 \end{matrix} \right)$$

Here, the monoclinic mirror plane is perpendicular to the crystallographic *b*-axis of the monoclinic cell of BaTiO_3_ which is parallel to $i=2$ crystal physics coordinate. Through rotating the unit cell by right angles, 4 such unit cells can be achieved, each associated with an area fraction labelled as below:

Domain 1: *a* || 1, *b* || 2, *c* || 3 (Area fraction: A_1_)

Domain 2: *a* || 2, *b* || -1, *c* || 3 (Area fraction: A_2_)

Domain 3: *a* || -1, *b* || -2, *c* || 3 (Area fraction: A_3_)

Domain 4: *a* || -2, *b* || 1, *c* || 3 (Area fraction: A_4_)

Here (*a*, *b*, *c*) denote the crystallographic axes of the monoclinic unit cell, $i=\left( 1,2,3 \right)$ denote the previously defined crystal physics coordinate system and the area fractions are constrained to A_1_ + A_2_+ A_3_ + A_4_ = 1.

For normal incidence ($\theta=0^{\circ}$)

From Equation 1, the induced nonlinear polarization can be calculated in the crystal coordinate system and can be transformed into lab coordinates (X, Y, Z). The resultant nonlinear polarization in the lab coordinates X (*p*-polarized) and Y (*s*-polarized) are tabulated below (Table S1):

**Table S4: *p* and *s*-polarization components for different domains**

|  | $P_{p}^{2\omega}$ | $P_{s}^{2\omega}$ |
| --- | --- | --- |
| Domain 1 | $d_{11}{cos}^{2}\varphi+d_{12}{sin}^{2}\varphi$ | $d_{26}sin2\varphi$ |
| Domain 2 | $d_{26}sin2\varphi$ | $d_{12}{cos}^{2}\varphi+d_{11}{sin}^{2}\varphi$ |
| Domain 3 | ${-(d}_{11}{cos}^{2}\varphi+d_{12}{sin}^{2}\varphi)$ | $-d_{26}sin2\varphi$ |
| Domain 4 | $-d_{26}sin2\varphi$ | ${-(d}_{12}{cos}^{2}\varphi+d_{11}{sin}^{2}\varphi)$ |

The effective SHG intensity can be calculated as follows:

$$I_{p}^{2\omega}\propto\left( P_{p}^{2\omega} \right)^{2}\propto\left( A_{1}P_{p, domain 1}^{2\omega}+A_{2}P_{p, domain 2}^{2\omega}+A_{3}P_{p, domain 3}^{2\omega}+A_{4}P_{p, domain 4}^{2\omega} \right)^{2}$$

This can be simplified to:

$I_{p}^{2\omega}\propto K_{1p}\left( {sin}^{2}\varphi+K_{2p}{cos}^{2}\varphi\right)^{2}+K_{3p}{sin}^{2}2\varphi+K_{4p}\left( {sin}^{2}\varphi+K_{2p}{cos}^{2}\varphi\right)sin2\varphi$ (3)

where,

$$K_{1p}=\delta A_{1}^{2}d_{12}^{2} K_{2p}=\frac{d_{11}}{d_{12}} K_{3p}=\delta A_{2}^{2}d_{26}^{2} K_{4p}=2\delta A_{1}\delta A_{2}d_{12}d_{26}$$

$\delta A_{1} = A_{1} - A_{3}$and $\delta A_{2} = A_{2} - A_{4}$. Similar expressions can also be derived for $I_{s}^{2\omega}$ with the following coefficients:

$$K_{1s}=\delta A_{2}^{2}d_{11}^{2} K_{2s}=\frac{d_{12}}{d_{11}} K_{3s}=\delta A_{1}^{2}d_{26}^{2} K_{4s}=2\delta A_{1}\delta A_{2}d_{11}d_{26}$$

For oblique incidence ($\theta=45^{\circ}$):

Equations for oblique incidence polarimetry involve more convoluted combinations of *d*-coefficients and area fractions, however they can be reduced to the same form as equation (3). The exact convolutions are listed below:

$$\begin{aligned} &K_{1p}=\frac{1}{8}\left[ 2\left( A_{1}+A_{3} \right)d_{32}+2\delta A_{1}d_{12}+2d_{31}(A_{2}+A_{4}) \right]^{2} \end{aligned}$$

$$\begin{aligned} K_{2p}=\frac{1}{2\sqrt{2K_{1p}}}\left[ \delta A_{1}\left( d_{11}+d_{13}-2d_{35} \right)+\left( A_{1}+A_{3} \right)\left( d_{31}+d_{33}-2d_{15} \right) \right.+\left( A_{2}+A_{4} \right)\left( d_{32}+d_{33}-2d_{24} \right] \end{aligned}$$

$$K_{3p}=\frac{\left[ \delta A_{2}\left( d_{26}-d_{35} \right)]^{2} \right.}{4}$$

$$K_{4p}=2\sqrt{K_{1p}K_{3p}}$$

$$K_{1s}=\frac{1}{4}\left( A_{2}-A_{4} \right)^{2}d_{11}^{2}$$

$$K_{2s}=\frac{1}{2\sqrt{K_{1s}}}\left( d_{12}+d_{13} \right)\left( A_{2}-A_{4} \right)$$

$$K_{3s}=\frac{1}{4}\left( \sqrt{2}A_{1}\left( d_{26}-d_{24} \right)-\sqrt{2}A_{3}\left( d_{26}+d_{24} \right)+\frac{d_{15}}{\sqrt{2}}\left( A_{4}-A_{2} \right) \right)^{2}$$

$$K_{4s}=2\sqrt{K_{1s}K_{3s}}$$

**
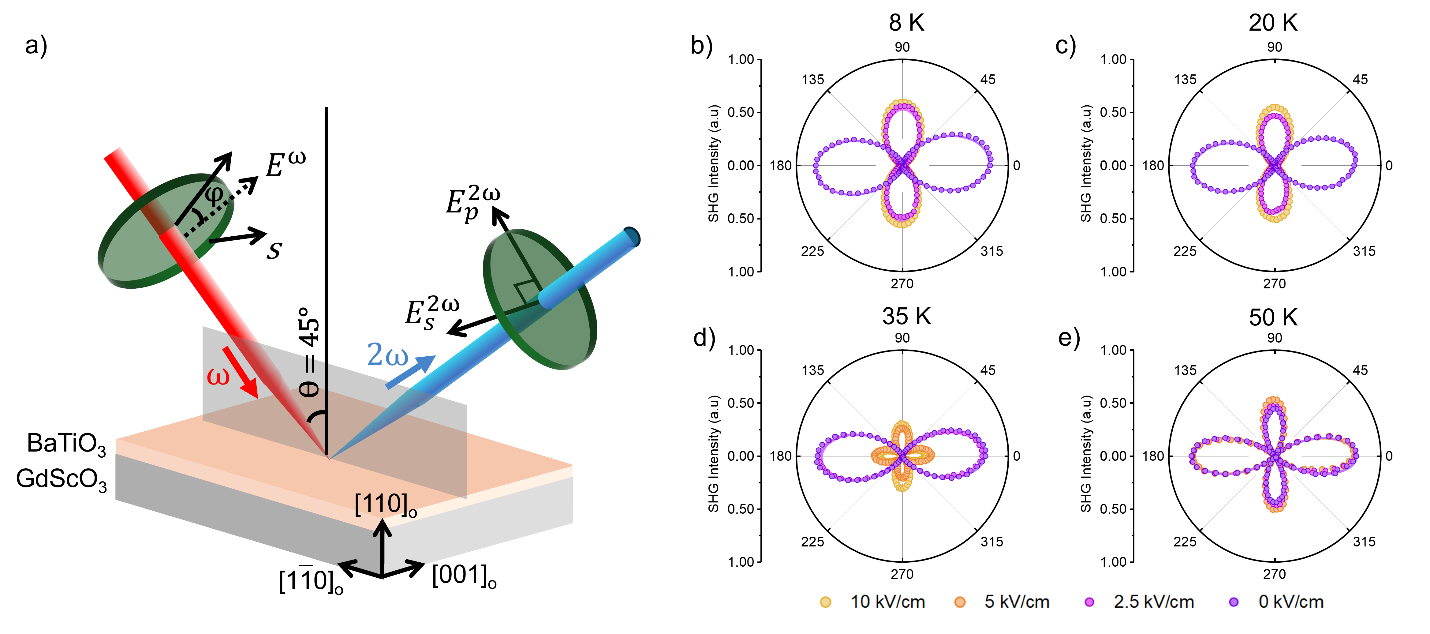
**

**Figure S20.** a) Schematic of the SHG experimental setup, where a fundamental probe at frequency *ω* generates second harmonic light at frequency 2*ω*. Crystallographic orientations of the orthorhombic GdScO_3_ substrate are indicated. b-e) Applied electric field dependent $I_{p}^{2\omega}$ polarimetry curves taken between 8 K and 50 K.


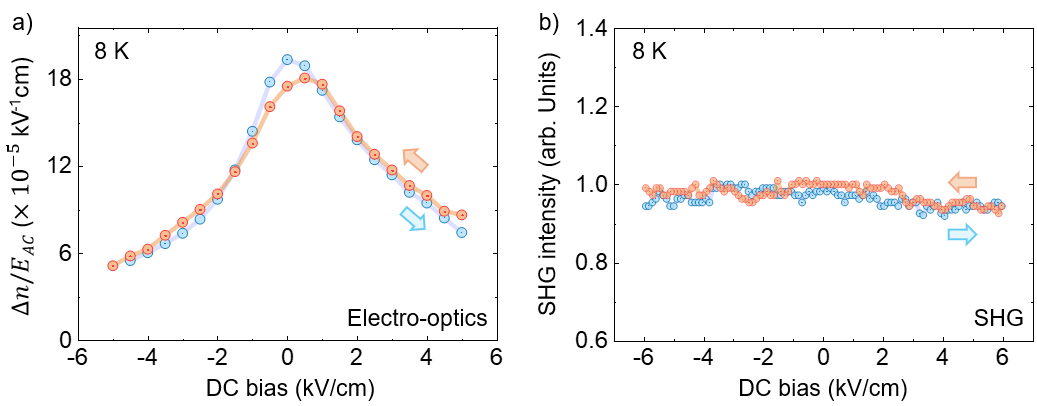


**Figure S21**: a) Hysteretic behavior of the electro-optic response of the BaTiO_3_/GdScO_3_ sample at low temperature obtained by sweeping the DC bias in the forward and reverse directions while using an AC electric field to measure the electric field-induced change in refractive index. b) SHG-DC bias loop measured at 8 K showing minimal hysteresis upon field sweep.

**Appendix A: Detailed expression of the free energy function and the associated parameters**

For BaTiO_3_, we use the cubic phase ($m\bar{3}m$) as our high symmetry reference state and employ an 8th-order landau expansion describe the relative stability of the lattice polarization compared to the cubic reference state. The coefficients used for this paper are adjusted from ^[14]^ and ^[15]^ to include the effect of cryogenic fluctuations and are given in **Table S5**.

**Table S5.** Coefficients in the thermodynamic free energy function and equation of motion for BaTiO_3_

| $g_{1111}^{LL}$ | $18.5\times{10}^{-2}\left( m^{4}/C^{2} \right)$ | $p_{1111}$ | $0.5328$ (Unitless) |
| --- | --- | --- | --- |
| $g_{1122}^{LL}$ | $2.5\times{10}^{-2}\left( m^{4}/C^{2} \right)$ | $p_{1122}$ | $0.1584$ (Unitless) |
| $g_{1212}^{LL}$ | $12.85\times{10}^{-2}\left( m^{4}/C^{2} \right)$ | $p_{1212}$ | $-0.432$ (Unitless) |
| $\mu_{e}$ | $35.5 \times{10}^{-23}\left( \frac{Kg}{m}\frac{m^{4}}{C^{2}} \right)$ | $\gamma_{e}$ | $3\times{10}^{-9}\left( \frac{Kg}{ms}\frac{m^{4}}{C^{2}} \right)$ |
| $a_{11}$ | $a_{0} T_{s} \left( \coth\left( \frac{T_{s}}{T} \right)-coth(\frac{T_{s}}{T_{c}} ) \right)$ | $B_{ij}^{e, ref}\left( T_{0} \right)$ | $0.2356$ (Unitless) |
| $T_{c}$ | $388K$ | $T_{0}$ | $398K$ |
| $T_{s}$ | 54 K | $a_{0}$ | $4.124\times{10}^{5}\left( \frac{J}{m^{3}} \frac{m^{4}}{{K C}^{2}} \right)$ |
| $a_{1111}$ | $-2.097\times{10}^{8}\left( \frac{J}{m^{3}} \frac{m^{8}}{C^{4}} \right)$ | $\alpha_{11}$ | $2.657\times{10}^{-5}\left( \frac{1}{K} \right)$ |
| $a_{1122}$ | $7.974\times{10}^{8}\left( \frac{J}{m^{3}} \frac{m^{8}}{C^{4}} \right)$ | $a_{11111111}$ | $3.863\times{10}^{10}\left( \frac{J}{m^{3}} \frac{m^{16}}{C^{8}} \right)$ |
| $a_{111111}$ | $1.294\times{10}^{9}\left( \frac{J}{m^{3}} \frac{m^{12}}{C^{6}} \right)$ | $a_{11111122}$ | $2.529\times{10}^{10}\left( \frac{J}{m^{3}} \frac{m^{16}}{C^{8}} \right)$ |
| $a_{111122}$ | $-1.95\times{10}^{9}$ $\left( \frac{J}{m^{3}} \frac{m^{12}}{C^{6}} \right)$ | $a_{11112222}$ | $1.637\times{10}^{10}\left( \frac{J}{m^{3}} \frac{m^{16}}{C^{8}} \right)$ |
| $a_{112233}$ | $-2.509\times{10}^{9}$ $\left( \frac{J}{m^{3}} \frac{m^{12}}{C^{6}} \right)$ | $a_{11112222}$ | $1.637\times{10}^{10}\left( \frac{J}{m^{3}} \frac{m^{16}}{C^{8}} \right)$ |
| $C_{11}$ | $1.78 \times{10}^{11}\mathrm{Pa}$ | $a_{11112233}$ | $1.367\times{10}^{10}\left( \frac{J}{m^{3}} \frac{m^{16}}{C^{8}} \right)$ |
| $C_{12}$ | $0.964 \times{10}^{11}\mathrm{Pa}$ | $C_{44}$ | $1.22 \times{10}^{11}\mathrm{Pa}$ |

References

[1] Every, A. G.; McCurdy, A. K. (Eds.). *Landolt-Börnstein: Numerical Data and Functional Relationships in Science and Technology, New Series, Group III* (Vol. 29a) (1992), Springer-Verlag, Berlin/Heidelberg

[2] Hellwege, K.-H.; Hellwege, A. M. (Eds.). *Landolt-Börnstein: Numerical Data and Functional Relationships in Science and Technology, New Series, Group III* (Vol. 16a) (1981), Springer-Verlag, Berlin/Heidelberg

[3] Uecker, R.; Velickov, B.; Klimm, D.; Bertram, R.; Bernhagen, M.; Rabe, M.; Albrecht, M.; Fornari, R.; Schlom, D. G. "Properties of rare-earth scandate single crystals (Re=Nd−Dy)", *Journal of Crystal Growth*, Vol. 310, No. 10, 2649–2658 (2008). https://doi.org/10.1016/j.jcrysgro.2008.01.019

[4] Matsubara, Y.; Takahashi, K. S.; Tokura, Y.; Kawasaki, M. "Single-crystalline BaTiO3 films grown by gas-source molecular beam epitaxy", *Applied Physics Express*, Vol. 7, No. 12, 125502 (2014). 10.7567/APEX.7.125502

[5] Coh, S.; Heeg, T.; Haeni, J. H.; Biegalski, M. D.; Lettieri, J.; Edge, L. F.; O’Brien, K. E.; Bernhagen, M.; Reiche, P.; Uecker, R.; Trolier-McKinstry, S.; Schlom, D. G.; Vanderbilt, D. "Si-compatible candidates for high-κ dielectrics with the Pbnm perovskite structure", *Physical Review B*, Vol. 82, No. 6, 64101 (2010). 10.1103/PhysRevB.82.064101

[6] Cook Jr., W. R. *3.3.17 Tetragonal, 4mm C4v: Datasheet from Landolt-Börnstein - Group III Condensed Matter · Volume 29B: ‘Piezoelectric, Pyroelectric, and Related Constants’ in SpringerMaterials*, (D. F. Nelson, Ed.) (Vol. 29B) (1993), Springer-Verlag Berlin Heidelberg. 10.1007/10049674_21

[7] Woollam, J. A.; Spectroscopic Ellipsometers, C. *CompleteEASE ® Data Acquisition and Analysis Software for Software Manual* (2014)

[8] Peiponen, K.-E.; Vartiainen, E. M. "Kramers-Kronig relations in optical data inversion", *Physical Review B*, Vol. 44, No. 15, 8301–8303 (1991). 10.1103/PhysRevB.44.8301

[9] De Sousa Meneses, D.; Malki, M.; Echegut, P. "Structure and lattice dynamics of binary lead silicate glasses investigated by infrared spectroscopy", *Journal of Non-Crystalline Solids*, Vol. 352, No. 8, 769–776 (2006). https://doi.org/10.1016/j.jnoncrysol.2006.02.004

[10] Fujiwara, H. *Spectroscopic Ellipsometry* (2007), John Wiley & Sons, Ltd, Chichester, UK. 10.1002/9780470060193

[11] Yariv, A.; Yeh, P. *Optical Waves in Crystals: Propagation and Control of Laser Radiation* (2002)

[12] Callister, W. D. *Materials Science and Engineering: An Introduction* (7th Edition.) (2007), John Wiley & Sons, Inc.

[13] Hazra, S.; Schwaigert, T.; Ross, A.; Lu, H.; Saha, U.; Trinquet, V.; Akkopru-Akgun, B.; Gregory, B. Z.; Mangu, A.; Sarker, S.; Kuznetsova, T.; Sarker, S.; Li, X.; Barone, M. R.; Xu, X.; Freeland, J. W.; Engel-Herbert, R.; Lindenberg, A. M.; Singer, A.; Trolier-McKinstry, S.; Muller, D. A.; Rignanese, G.-M.; Salmani-Rezaie, S.; Stoica, V. A.; Gruverman, A.; Chen, L.-Q.; Schlom, D. G.; Gopalan, V. "Colossal Strain Tuning of Ferroelectric Transitions in KNbO3 Thin Films", *Advanced Materials*, Vol. 36, No. 52, 2408664 (2024). https://doi.org/10.1002/adma.202408664

[14] Ross, A.; Ali, M. S. M. M.; Saha, A.; Zu, R.; Gopalan, V.; Dabo, I.; Chen, L.-Q. "Thermodynamic theory of linear optical and electro-optical properties of ferroelectrics", *Physical Review B*, Vol. 111, No. 8, 85109 (2025). 10.1103/PhysRevB.111.085109

[15] Li, Y. L.; Cross, L. E.; Chen, L. Q. "A phenomenological thermodynamic potential for BaTiO3 single crystals", *Journal of Applied Physics*, Vol. 98, No. 6, 064101 (2005). 10.1063/1.2042528
